# Effect of Dietary Fiber on the Composition of the Murine Dental Microbiome

**DOI:** 10.3390/dj7020058

**Published:** 2019-06-01

**Authors:** Lea Sedghi, Craig Byron, Ryan Jennings, George E. Chlipala, Stefan J. Green, Laura Silo-Suh

**Affiliations:** 1 Department of BioMedical Sciences, School of Medicine, Mercer University, Macon, GA 31207, USA; Lea.Maryam.Sedghi@live.mercer.edu; 2 Department of Biology, Mercer University, Macon, GA 31207, USA; Byron_cd@mercer.edu; 3 Department of Biology, Frostburg State University, Frostburg, MD 21532-2303, USA; rdjennings@frostburg.edu; 4 Research Resources Center, University of Illinois at Chicago, Chicago, IL 60612-3748, USA; gchlip2@uic.edu (G.E.C.); greendna@uic.edu (S.J.G.)

**Keywords:** dental microbiome, dietary sugar, dietary fiber, 16S, biofilm

## Abstract

The oral cavity houses a diverse consortium of microorganisms, heavily influenced by host diet, that can mediate dental health and disease. While the impact of dietary carbohydrates to the dental microbiome has been well-documented, the effect of fiber as a mechanical influence on the dental microbiome is unexplored. We performed 16S rRNA gene analysis to investigate the response of the dental microbiome to the presence of increased fiber in terms of microbial taxonomic abundance and diversity. Dental microbial community structure was significantly different in mice fed a diet supplemented with increased fiber and/or sugar. Fiber significantly affected measures of beta diversity at the phylum and genus levels, and a strong interactive effect on alpha diversity was observed between sugar and fiber at the phylum level. The addition of fiber also induced significant variation in relative taxonomic abundance. This study demonstrates that fiber can promote significant variations in the mouse dental microbiome.

## 1. Introduction

The oral cavity consists of diverse structures and tissue surfaces that harbor unique microbial communities which are impacted daily by mechanical and chemical influences, including variation in diet [1]. The composition of microbial communities subsequently impacts states of oral health and disease [2]. For example, diets rich in carbohydrates are associated with microbial populations related to increased incidence of periodontal disease and dental caries [3,4]. Arguably, the oral microbiome has been shaped by sociocultural changes that accompanied our evolutionary history, especially following the onset of the Neolithic and Industrial eras [5,6,7]. Specifically, the advent of agriculture has been implicated as a causative factor in the emergence of dental disease [8,9], presumably due to a significant increase in carbohydrate intake [7,10,11]. In addition to nutritional changes, substantial changes in dietary fiber also resulted from abandonment of the pre-agricultural diet. Many staples of the Western diet, and specifically the U.S diet, such as refined sugars, vegetable oils, and dairy products, are devoid of fiber [6].

The teeth are the only inherent non-shedding surfaces within the human body, and the stagnancy of these surfaces provides unique opportunities for extensive microbial biofilm accumulation [12,13]. As a result, modern dental practice suggests daily mechanical disruption of the dental biofilm via the manual use of a toothbrush [14]. Fibrous pre-agricultural diets may have prevented dental biofilm formation by physically disrupting them as a natural outcome of oral processing, mastication, and ingestion of these foods. Much of the evidence for fiber functioning as a mechanical force on dental calculus comes from animal studies, although several human studies support the indication [15,16,17,18]. While the benefits of fiber to oral health have been noted [19,20], an understanding of the impact of fiber at the microbial scale, and to the dental surfaces specifically, is unrecognized.

Comprehensive metagenomic studies of the murine or rat dental microbiome are lacking. Some of the earliest studies that investigated the oral microbiome of mice used cultivation methods that identified only a small fraction of the bacteria we now know inhabit this niche [21,22,23]. More recent studies using high-throughput sequencing methods have examined the dorsal surface of the rat tongue [24], the gingival tissues and oral mucosal surfaces of mice [25], and excised pieces of tongue, palate, and incisors from mice [26]. In general, these studies determined that the mouse and rat oral microbiomes are less diverse than human microbiomes. Moreover, the animal microbiomes stabilize once the animals are adapted to the animal facility. Both of these factors may be related to the initial colonization of the animals under controlled laboratory settings and consumption of a constant diet. This was supported by a current study that used human salivary microbiota to colonize the oral cavities of gnotobiotic mice [27]. In this study, the recipient mice maintained an oral microbiota that was highly representative of the donor microbiota. Notably, the study also indicated that the donor oral bacteria colonized the mouse gut. Comprehensive studies on the effect of diet on the oral microbiome are also lacking, but the few existing studies indicate that diet also influences the oral microbiome of humans and rats [28,29]. Despite the advances these studies have made in providing a greater characterization of the oral microbiome, these studies do not specifically characterize the dental microbiome of mice. The surfaces within the mouth differ widely from one location to another, and the nature of the dental surfaces makes this stratum unique within the oral cavity for microbial growth [30,31]. Thus, it is important to characterize the dental microbiome in isolation from other sites within the oral cavity.

This study used 16S rRNA gene analysis to identify the influence of fiber, alone and in the presence of sugar, on microbial population structure within the dental biofilm. Using an in vivo murine model, dental surfaces of mice were isolated to study the impact of fiber on microbial abundance and diversity. This preliminary study indicates that dietary texture impacts the composition of the dental microbiome. Moreover, we also introduce a novel whole-jaw extraction technique to isolate the dental biofilm in its entirety, and in isolation from other sites within the oral cavity.

## 2. Material and Methods

### 2.1. Dental Microbiome Sample Collection and Ethical Statement

Animal usage was approved by Mercer University’s Institutional Animal Care and Use Committee (IACUC #A1609015) on 23 September 2016. Three-week-old female mice (*N* = 28) of the ICR/CD-1 strain (Charles River Labs, Wilmington, MA, USA) were housed in standard shoebox rodent housing throughout the duration of the experiment. Mice were randomized into four groups (*N* = 7 each) that contrast with respect to dietary fiber and sugar. A control diet using 2-ounce cups of DietGel 76A (Clear H_2_O, Westbrook, ME, USA) served as the base for all groups. This product delivers essential nutrition and hydration for lab rodents. The control diet was supplemented with sucrose (6.5 g/2-oz) or lignin (4.2 g/2-oz) to obtain the high-sugar (Sugar) and high-fiber (Fiber) diets, respectively. The fourth diet (Sugar + Fiber) contained both sucrose (6.5 g/2-oz) and lignin (4.2 g/2-oz). Prior to instituting the diet regimens, all mice were fed the base diet for 10 days.

Groups of three or four mice were caged by diet without substrates on which to chew (i.e., typical wire top lids or water bottle sipper tubes) and kept under controlled environmental conditions (temperature 26  ±  0.5 °C, 12/12-h light/dark cycle) with free access to feed. Mouse cages were cleaned and refreshed weekly over the course of the study. A small amount of litter (~15 mL or 1 Tablespoon) from each cage was mixed together during the time of cage cleaning. This intermixed old litter was distributed into the new shoebox cages (15 mL each). Therefore, any changes in oral microbial communities could be attributed to dietary differences alone, as opposed to differences in habitat-specific microbial communities. After 60 days of a specific dietary regimen, mice were euthanized using CO_2_ overdose. The lower jaw of each specimen was micro-dissected and cleaned of most muscle, epithelia, and connective tissues using sterile methods under controlled airflow. Careful attention was given not to disturb the dental surface. Jaws were stored at −80 °C until extraction.

### 2.2. Characterization of Dental Microbiome

DNA was isolated from the extracted jaws using the DNeasy PowerBiofilm Kit (Qiagen Inc., Qiagen, MD, USA). The entire jaw was added to the extraction tube for processing. Extractions were carried out according to the manufacturer’s instructions. Genomic DNA was PCR-amplified with primers CS1_515F and CS2_806R, using a two-stage “targeted amplicon sequencing (TAS)” protocol, as described previously [32]. After the second-stage amplification, libraries were pooled and subject to size selection (350–450 bp) using a BluePippin device (Sage Science, Beverly, MA, USA). Size selection was used to remove additional amplification products, including host mitochondrial 16S rRNA gene amplicons and host 18S rRNA gene amplicons which were generated due to high host-to-microbe DNA ratios in samples. The pooled libraries, with a 20% phiX spike-in, were loaded onto an Illumina MiniSeq mid-output flow cell (2 × 153 paired-end reads). Based on the distribution of reads per barcode, the amplicons were re-pooled to generate a more balanced distribution of reads, and subject to another PippinPrep size selection. The re-pooled libraries were loaded onto a MiSeq V2 mid-output flow cell to generate final data. Library preparation, pooling, and sequencing were performed at the University of Illinois at Chicago Sequencing Core (UICSQC). Raw sequence data were processed using a QIIME workflow with GreenGenes v13 8 [33], as described previously, with minor modifications. The modifications included discarding sequences shorter than 225 bases, and when performed, rarefaction to a depth of 25,000 sequences per sample. The final outputs of the QIIME pipeline were biological observation matrices (BIOMs; [34]) at taxonomic levels from phylum to genus.

### 2.3. Alpha Diversity Analyses

Shannon indices were calculated on rarefied data with default parameters in the ‘R’ environment using the vegan software package [35]. Shannon indices were modelled with dietary factors using a generalized linear model (GLM), assuming a Gaussian distribution. Significance of the model (ANOVA) was determined using the *F* test. Post-hoc, pairwise tests were performed using the Mann–Whitney test. Plots were generated in R using the ggplot2 library [36].

### 2.4. Beta Diversity Analyses

Bray–Curtis indices were calculated with default parameters in R using the vegan library. Prior to analysis, data were log_10_(*x* + 1) transformed. The resulting dissimilarity indices were modelled and tested for significance with the dietary groups using the ADONIS test. Additional comparisons of each dietary factor, separately, were also performed using ANOSIM. Plots were generated in R using the ggplot2 library [36]. A heatmap was generated in the software package Primer7 (Primer-E, Plymouth, UK).

### 2.5. Differential Analysis of Microbial Taxa

Differential analyses of taxa, as compared with dietary factors (i.e., sugar and fiber), were performed using the software package edgeR on raw sequence counts [37]. Prior to analysis the data were filtered to remove any sequences that were annotated as chloroplast or mitochondria in origin, as well as removing taxa that accounted for less than 0.1% of the total sequence counts. Data were normalized using a trimmed mean of M-values (TMM). Normalized data were then fit using a negative binomial generalized linear model, using Sugar and Fiber covariates, and statistical tests were performed using a likelihood ratio test. Adjusted *p* values (*q* values) were calculated using the Benjamini-Hochberg false discovery rate (FDR) correction [38]. Significant taxa were determined based on an FDR threshold of 5% (0.05).

### 2.6. Data Archive

Raw sequence data have been deposited in NCBI Short Read Archive under accession numbers SRR8582882-SRR8582864.

## 3. Results

### 3.1. Experimental Setup

In order to study the effect of fiber as an abrasive component to the murine dental microbiome, a base diet was supplemented with lignin to obtain a high-fiber diet. Lignin is a non-digestible insoluble fiber found in plant foods. Most microbes do not readily metabolize lignin and the microbes that can metabolize lignin have not been identified within the oral cavity [39]. Therefore, lignin functions here solely as a dietary texture component without contributing a direct metabolic influence. The control base diet (Control) was amended with lignin alone (Fiber), with sucrose alone (Sugar), and with lignin and sucrose together (Sugar + Fiber).

Two key limitations in comprehensive sampling of the dental microbiome have been isolation of the intact dental biofilm specifically and in its entirety. To overcome these limitations, we removed the entire mandible at the temporomandibular joint for genomic DNA extraction. The isolation of the dental surface microbiome from other intraoral microbial communities is important because each surface within the mouth harbors distinct microbial communities [31,40,41].

### 3.2. Diet-Induced Changes in Microbial Community Structure

**Figure 1:**
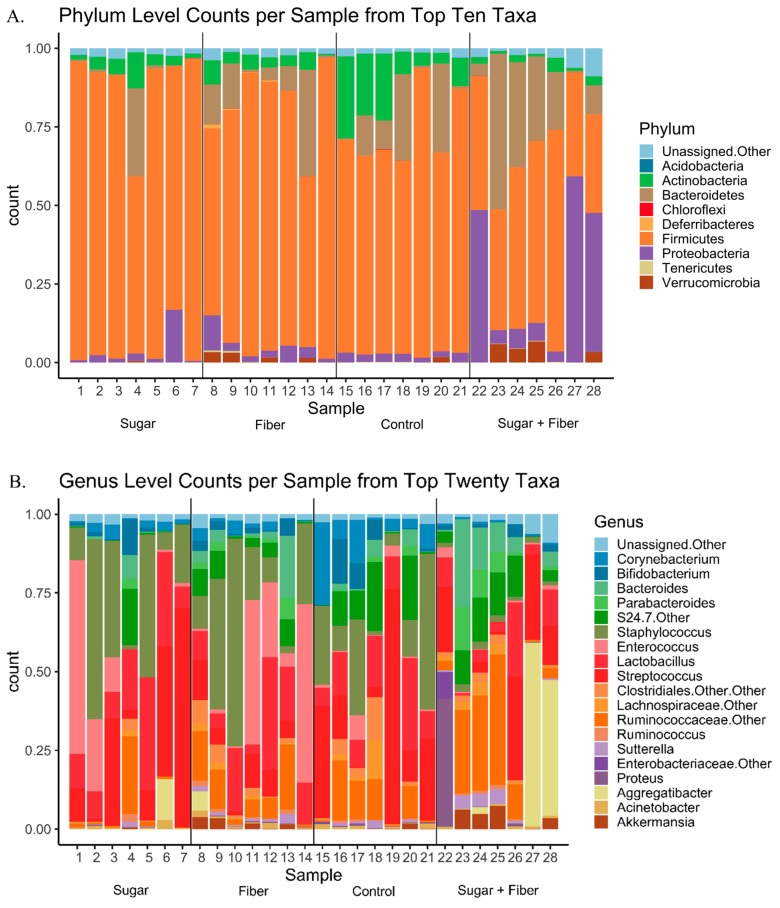
Stacked bar charts of major taxon relative abundance by sample. Sample numbers are associated with the following diet groups: 1–7 (Sugar), 8–14 (Fiber), 15–21 (Control), 22–28 (Sugar + Fiber). (**A**) Taxonomic level of phylum; (**B**) Taxonomic level of genus.

The microbial community structure of dental surfaces was profiled in mice fed varying diets, and the dietary variables significantly affected the microbial composition (diversity and abundance) of mouse host-associated oral-mandibular samples. The V4 variable region of the microbial 16S rRNA gene was amplified from genomic DNA derived from whole jaw extractions. Sequencing of 16S rRNA gene amplicons produced 700,000 sequences with 25,000 sequences per mouse, post-quality filtering. Seven subjects comprised each feeding group (28 mice in total). The mouse dental microbiome, across all treatments, was dominated by sequences from bacteria from four phyla, including: Firmicutes (70.0%), Bacteroidetes (11.7%), Proteobacteria (8.6%), and Actinobacteria (5.9%) (Figure 1A). Bacteria from other phyla, including Acidobacteria, Chloroflexi, Deferribacteres, Tenericutes, and Verrucomicrobia, were also detected, but the relative abundance of these taxa never exceeded 13% in any sample. At the taxonomic level of genus, the 20 most abundant bacterial genera represented >84% of all sequences from all samples (Figure 1B).

**Figure 2:**
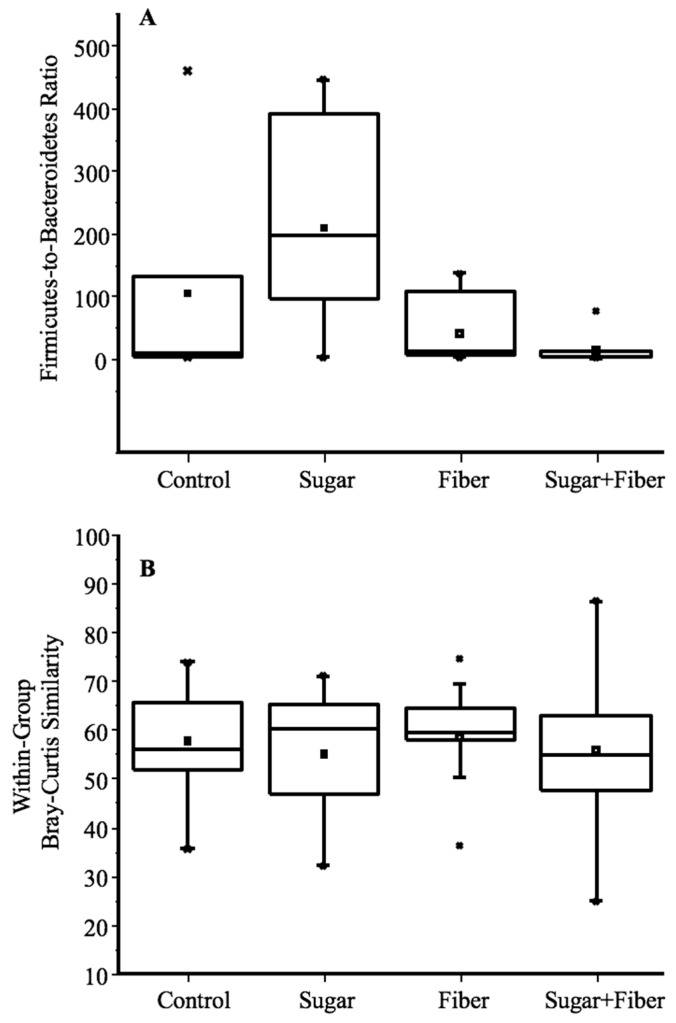
(**A**) Effect of treatment on Firmicutes-to-Bacteroidetes (*F/B*) ratios in murine dental microbiome. The *F/B* ratio was significantly different between ‘Sugar’ and ‘Sugar + Fiber’ treatments (ANOVA, Tukey’s Test, *p* = 0.028). (**B**) Box plot of within-group Bray–Curtis similarity distributions for each treatment group. Bray–Curtis similarity values were calculated using a genus-level biological observation matrix (BIOM). Within-group Bray–Curtis similarity of replicates was not significantly different between treatments (one-way ANOVA; *p* = 0.788).

A ratio of Firmicutes-to-Bacteroidetes (*F/B*) was calculated for each sample, and differences between groups were assessed using ANOVA and Tukey’s tests (Figure 2A). Tukey’s tests indicated that the *F/B* ratio was significantly different only between treatments of Sugar alone and Sugar + Fiber (*p* = 0.028). Except for one replicate (*F/B* ratio of 2.0), the *F/B* ratios within the Sugar treatment were very high, ranging from 93.4 to 444.8. Conversely, the *F/B* ratios within the Sugar + Fiber treatment were low, ranging from 0.8 to 3.8, with one outlier (*F/B* ratio of 75.3). The relative abundance of bacteria from the phylum Proteobacteria was generally low across all treatments, though several individual mice within the Sugar + Fiber treatment had a very high relative abundance of individual proteobacterial genera, including *Proteus* and *Aggregatibacter* (38.7% to 57.4% for 3 of 7 replicates). In all other samples, Proteobacterial sequences never exceed 13%, and were generally much lower (<3%).

**Figure 3:**
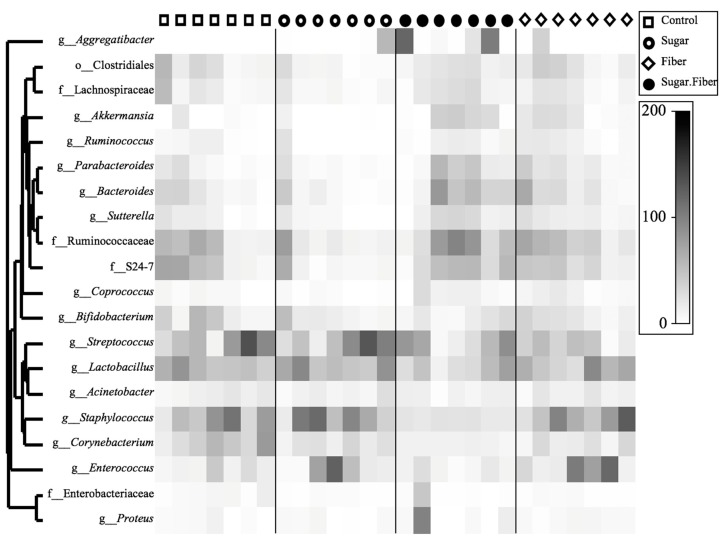
One-way clustered heatmap of genus-level abundance of the 20 most abundant microbial taxa. Data are square root-transformed and are based on sequence datasets rarefied to 25,000 sequences per sample.

The shift in the *F/B* ratio, as seen above, was largely due to the higher relative abundance in the Sugar + Fiber treatment of three taxa within the phylum Bacteroidetes, including the genera *Bacteroides* and *Parabacteroides*, and the family ‘S24-7′. Overall, within each treatment group, microbial community structure varied substantially between replicates, with Bray–Curtis similarity scores ranging from 0.25 to 0.86 (Figure 2B). The within-group similarity of replicates was not, however, significantly different between treatments (ANOVA, *p* = 0.79; Figure 2B). In addition, the relative abundance of *Staphylococcus*, *Enterococcus*, *Lactobacillus,* and *Streptococcus* was substantially higher in mice from the Sugar only treatment, relative to the mice from the Sugar + Fiber treatment (Figure 3).

**Figure 4:**
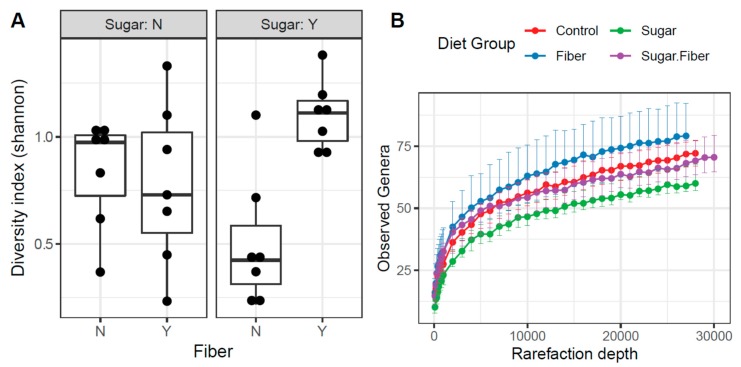
(**A**) Interactive effects of Sugar and Fiber on alpha diversity (Shannon Index) of murine dental microbiomes. The Sugar + Fiber group demonstrated an increase in community diversity compared to the Sugar group alone. N = absence of additive and Y = presence of additive. (**B**) Rarefaction curves display median number of observed genera for each dietary group. Rarefaction was performed using genus-level summary at depths of 100 to 30,000 with steps of 100 from 100 to 1,000 counts, and then steps of 1000 from 1000 to 30,000.

Alpha diversity analyses were performed to determine if the general diversity of the murine dental microbiome was affected by dietary treatment. A significant effect caused by treatments on the Shannon index (a combination of microbial richness and evenness) was observed at the phylum level but not the genus level. At the taxonomic level of phylum, the Shannon index was significantly higher in the presence of fiber (*F* test; *p* = 0.021) but not sugar (*F* test; *p* = 0.978). A significantly increased phylum-level alpha diversity in the presence of sugar was observed when combined with fiber as a statistical interaction (*F* test; *p* = 0.006). Pairwise testing between dietary groups revealed a significant change only in alpha diversity between the Sugar + Fiber and Sugar groups (Mann–Whitney *p* < 0.01). Furthermore, box and whisker plot comparisons revealed that the Sugar + Fiber group demonstrated an increase in community diversity when compared to the Sugar group alone, which had the lowest observed alpha diversity (Figure 4).

Analyses of species richness and evenness were performed to determine the factors primarily driving the changes in the Shannon Index values. At the taxonomic level of phylum, the presence of fiber significantly increased community richness (GLM; *Pr* > *F* = 0.013). In contrast, the presence of sugar did not (GLM; *Pr* > *F* = 0.596), nor did the interaction of sugar and fiber (GLM; *Pr* > *F* = 0.790). The Kruskal–Wallis test is consistent with this observation, showing that fiber had a significant effect on community richness (*p* = 0.005) and sugar did not (*p* = 0.849). The interaction of sugar and fiber significantly increased community richness compared to the presence of sugar alone, as revealed by pairwise comparison of these groups (*p* = 0.047). This demonstrated that in the presence of sugar, fiber significantly impacted species richness. At the genus level, the presence of sugar had a significant effect on community richness (GLM; *Pr* > *F* = 0.002), as did the presence of fiber (GLM; *Pr* > *F* = 0.013), but not by the interaction of Sugar + Fiber (GLM; *Pr* > *F* = 0.752). The Kruskal–Wallis analysis was consistent with the observation for Sugar (*p* = 0.005) and Fiber (*p* = 0.027). The Control group had significantly greater community richness than the Sugar diet alone, as revealed by pairwise comparison of these groups (*p* = 0.009). A near-significant increase in community richness in the Fiber group was observed in pairwise comparisons of the control and Fiber groups (*p* = 0.055).

At the taxonomic level of phylum, beta diversity analyses demonstrated that microbial community dissimilarity was significantly associated with the presence or absence of fiber (ADONIS R^2^ = 0.321; *p* = 0.002) (Table 1). In contrast, Sugar and the interaction between Sugar + Fiber were not significant. Additional analysis of microbial dissimilarity for Fiber corroborated this finding (ANOSIM R = 0.382; *p* = 0.001), while the results for Sugar were not significant (ANOSIM R = 0.033; *p* = 0.232) (Table 1). The same effect was observed at the taxonomic level of genus.

### 3.3. Taxonomic Abundance of Dietary Groups

An omnibus analysis identified four significant changes in the relative abundance of genera between diets. This included an increase in the relative abundance of *Aggregatibacter* in the presence of sugar (*q* < 0.001) and fiber (*q* < 0.05), while the relative abundance of *Streptococcus* was significantly affected by fiber (*q* < 0.05). In addition, a significant decrease in the relative abundance of *Enterococous* was observed for the interaction between sugar and fiber in the study (*q* < 0.01), while the relative abundance of the genus *Staphylococcus* decreased significantly with the Sugar + Fiber diet, which was consistent with the observed statistical interaction between independent variables sugar and fiber (*q* < 0.01) (Supplementary materials).

In addition, significant differences were observed for pairwise comparisons between each dietary group. Many genera having significant pairwise comparisons (*q* < 0.05) were identified in the comparison of the Sugar + Fiber, and Sugar diets. These genera included *Aggregatibacter*, Enterobacteriaceae: Other, *Corynebacterium*, *Enterococcus*, *Dorea*, *Blautia*, *Coprococcus*, *Akkermansia*, *Parabacteroides*, *Bacteroides*, Planococcae, *Proteus*, *Staphylococcus*, rc4-4, and *Aldercruzia* (Supplementary materials).

## 4. Discussion

This comparative analysis describes the response of the murine dental microbiome to the addition of high-sugar and/or high-fiber to a base diet. While consumption of excess carbohydrates and sugar can have detrimental effects on dental health and the oral microbiome [42], the impact of fiber has been less well-characterized. This study demonstrates that a dissimilar microbial community was established in the presence of fiber compared to the presence of sugar, or the interaction of sugar and fiber. The presence of additional fiber in the diet promoted significant changes in dental microbiome beta diversity at the taxonomic level of genus. At the taxonomic level of phylum, significant variations in microbial community alpha diversity were observed between the Sugar treatment and the Sugar + Fiber treatment. These treatments had large significant differences in the ratio of Firmicutes-to-Bacteroidetes (*F/B*), and these alterations were significant when calculating alpha diversity indices, such as the Shannon index or Pielou’s evenness. Prior studies have shown that diets high in added sugars are associated with decreased microbial diversity and increased levels of caries-associated bacteria [42,43,44]. This is consistent with the high *F/B* ratio in the Sugar treatment in this study, leading to low diversity measures. Conversely, in the presence of fiber, the added sugar did not lead to a dramatic shift in *F/B* ratio but did lead to a significantly higher alpha diversity due to increased evenness of the most abundant taxa.

In the Sugar treatment, the dental microbial community was largely dominated by bacteria from the genera *Streptococcus*, *Staphylococcus*, *Lactobacillus*, and *Enterococcus*. Members of these genera have been previously associated with human pathogenesis. As the levels of these potentially pathogenic taxa decreased in the Sugar + Fiber treatment, the relative abundance of potentially beneficial taxa, such as *Akkermansia mucinophila*, increased. Dietary fiber stimulates *Akkermanisa* populations in the gut, and their proliferation has been associated with reduced inflammation, metabolic disorders, obesity, and diabetes [45,46]. *Akkermansia* spp. have rarely been identified in the oral cavity and never in high abundance [47]. To our knowledge, this is the first study to show that *Akkermansia* populations in the oral cavity can also be affected by diet, and specifically by fiber.

The interaction of Sugar and Fiber unexpectedly showed signs of a potentially healthier oral microbiome compared to the other three dietary regimens. At the phylum level, this included increased alpha diversity and a significant increase in Proteobacteria, which have been previously noted to comprise a healthy oral microbiome [48]. We also observed changes in the relative abundance of bacteria from the genera *Corynebacterium* in the pairwise comparison of the Sugar + Fiber diet to the Sugar diet, demonstrating that fiber had a significant impact on decreasing relative abundance of this taxon in the presence of sugar. *Corynebacterium* is notable for its extensive involvement in dental biofilm formation [49]. Early colonizers such as *S. mitis* and *Actinomyces* occupy the base of such biofilms, and *Corynebacterium* attaches to these early colonizers and provides extended filaments throughout the structure, upon which additional bacterial attachment sites are created to produce “hedgehog structures”. Therefore, the loss of *Corynebacterium* may undermine the formation of hedgehog structures that are the cornerstone of dental biofilm communities.

Further investigations will be needed to determine if diet-induced alterations in the oral cavity precede and influence colonization in the gut. Non-digestible fibers provide metabolic substrates for colonic bacteria, here we demonstrated that such fibers also alter the murine dental microbiota, possibly leading to a greater understanding of the association of oral health to gastrointestinal and systemic health. We speculate that dietary fiber acts as a mechanical force on the dental surfaces to aid in the shedding of the dental microbial biofilm.

The dental model presented in this study can be used to develop a comprehensive and systematic profiling of the microbiome specific to the dental surfaces and to understand microbial associations in dental and periodontal disease. We also note the development of a whole mandible extraction and sequencing protocol, allowing for robust analysis of the murine dental microbiome. Furthermore, due to substantial variability within treatment groups, further studies with greater numbers of mice should be employed, and a greater number of pairwise comparisons may emerge as significant.

## Figures and Tables

**Table 1:** ADONIS and ANOSIM analyses of beta diversity at the phylum and Genus levels. ANOSIM analysis revealed a significant change in Beta diversity at the phylum and genus levels for the fiber diet alone. ADONIS analysis also revealed that beta diversity was significantly impacted by the presence of fiber at both levels. NS, non-significant. NA, non-applicable.

Beta Diversity at the Phylum Level					Beta Diversity at the Genus Level			
ADONIS			ANOSIM		ADONIS		ANOSIM	
Factor	*R* ^2^	*Pr* (>*F*)	*R*	*p*	*R* ^2^	*Pr* (>*F*)	*R*	*p*
Sugar	0.03	NS	0.03	NS	0.03	0.331	0.02	0.225
Fiber	0.32	<0.01	0.38	<0.01	0.18	< 0.01	0.20	<0.01
Sugar + Fiber	0.04	NS	NA	NA	0.07	0.082	NA	NA

## Supplementary Materials

The following are available online at https://www.mdpi.com/2304-6767/7/2/58/s1, Supplemental Table S1: Taxonomic Abundance of Dietary Groups.
