# Supplementary material for: Effect of Dietary Fiber on the Composition of the Murine Dental Microbiome
Source: Dent J (Basel). 2019 Jun 1;7(2):58. doi: 10.3390/dj7020058 (PMC6630570; doi:10.3390/dj7020058)
Supplement: Supplementary file 1 [file dentistry-07-00058-s001.zip › dentistry-479857-supplementary.docx]

**Supplementary Materials: Effect of Dietary Fiber on the Composition of the Murine Dental Microbiome**

**Lea Sedghi, Craig Byron, Ryan Jennings, George E. Chlipala, Stefan J. Green, and Laura Silo-Suh**

Supplementary Information

Contents

[1 Differential analysis at genus level 3](#_Toc2593349)

[1.1 Omnibus tests 3](#_Toc2593350)

[1.2 Pairwise comparison of Sugar N, Fiber Y vs. Sugar N, Fiber N 4](#_Toc2593351)

[1.3 Pairwise comparison of Sugar Y, Fiber N vs. Sugar N, Fiber N 5](#_Toc2593352)

[1.4 Pairwise comparison of Sugar Y, Fiber Y vs. Sugar N, Fiber Y 6](#_Toc2593353)

[1.5 Pairwise comparison of Sugar Y, Fiber Y vs. Sugar Y, Fiber N 8](#_Toc2593354)

[2 Differential analysis at family level 10](#_Toc2593355)

[2.1 Omnibus tests 10](#_Toc2593356)

[2.2 Pairwise comparison of Sugar N, Fiber Y vs. Sugar N, Fiber N 11](#_Toc2593357)

[2.3 Pairwise comparison of Sugar Y, Fiber N vs. Sugar N, Fiber N 12](#_Toc2593358)

[2.4 Pairwise comparison of Sugar Y, Fiber Y vs. Sugar N, Fiber Y 13](#_Toc2593359)

[2.5 Pairwise comparison of Sugar Y, Fiber Y vs. Sugar Y, Fiber N 14](#_Toc2593360)

[3 Differential analysis at order level 15](#_Toc2593361)

[3.1 Omnibus tests 15](#_Toc2593362)

[3.2 Pairwise comparison of Sugar N, Fiber Y vs. Sugar N, Fiber N 16](#_Toc2593363)

[3.3 Pairwise comparison of Sugar Y, Fiber N vs. Sugar N, Fiber N 17](#_Toc2593364)

[3.4 Pairwise comparison of Sugar Y, Fiber Y vs. Sugar N, Fiber Y 18](#_Toc2593365)

[3.5 Pairwise comparison of Sugar Y, Fiber Y vs. Sugar Y, Fiber N 19](#_Toc2593366)

[4 Differential analysis at class level 20](#_Toc2593367)

[4.1 Omnibus tests 20](#_Toc2593368)

[4.2 Pairwise comparison of Sugar N, Fiber Y vs. Sugar N, Fiber N 21](#_Toc2593369)

[4.3 Pairwise comparison of Sugar Y, Fiber N vs. Sugar N, Fiber N 21](#_Toc2593370)

[4.4 Pairwise comparison of Sugar Y, Fiber Y vs. Sugar N, Fiber Y 22](#_Toc2593371)

[4.5 Pairwise comparison of Sugar Y, Fiber Y vs. Sugar Y, Fiber N 22](#_Toc2593372)

[5 Differential analysis at phylum level 23](#_Toc2593373)

[5.1 Omnibus tests 23](#_Toc2593374)

[5.2 Pairwise comparison of Sugar N, Fiber Y vs. Sugar N, Fiber N 23](#_Toc2593375)

[5.3 Pairwise comparison of Sugar Y, Fiber N vs. Sugar N, Fiber N 24](#_Toc2593376)

[5.4 Pairwise comparison of Sugar Y, Fiber Y vs. Sugar N, Fiber Y 24](#_Toc2593377)

[5.5 Pairwise comparison of Sugar Y, Fiber Y vs. Sugar Y, Fiber N 24](#_Toc2593378)

# Differential Analysis at Genus Level

## Omnibus Tests

| Taxon | Sugar | | Fiber | | Sugar::Fiber* | |
| --- | --- | --- | --- | --- | --- | --- |
|  | *p* | *q*^†^ | *p* | *q*^†^ | *p* | *q*^†^ |
| k__Bacteria;p__Proteobacteria;c__Gammaproteobacteria;o__Pasteurellales;f__Pasteurellaceae;g__Aggregatibacter | 1.09 × 10^−5^ | 3.28 × 10^−4^ | 1.19 × 10^−3^ | 0.02 | 0.16 | 0.27 |
| k__Bacteria;p__Firmicutes;c__Bacilli;o__Lactobacillales;f__Streptococcaceae;g__Streptococcus | 0.76 | 0.82 | 1.47 × 10^−3^ | 0.02 | 0.06 | 0.17 |
| k__Bacteria;p__Proteobacteria;c__Gammaproteobacteria;o__Enterobacteriales;f__Enterobacteriaceae;Other | 0.02 | 0.10 | 2.78 × 10^−3^ | 0.03 | 3.80 × 10^−6^ | 1.14 × 10^−4^ |
| k__Bacteria;p__Actinobacteria;c__Actinobacteria;o__Actinomycetales;f__Corynebacteriaceae;g__Corynebacterium | 0.08 | 0.21 | 8.33 × 10^−3^ | 0.06 | 0.92 | 0.94 |
| k__Bacteria;p__Firmicutes;c__Bacilli;o__Lactobacillales;f__Enterococcaceae;g__Enterococcus | 0.02 | 0.10 | 0.02 | 0.10 | 3.48 × 10^−5^ | 5.22 × 10^−4^ |
| k__Bacteria;p__Firmicutes;c__Clostridia;o__Clostridiales;f__Lachnospiraceae;Other | 0.02 | 0.10 | 0.03 | 0.13 | 0.01 | 0.06 |
| k__Bacteria;p__Firmicutes;c__Clostridia;o__Clostridiales;f__Lachnospiraceae;g__Dorea | 0.07 | 0.21 | 0.06 | 0.28 | 0.33 | 0.41 |
| k__Bacteria;p__Firmicutes;c__Clostridia;o__Clostridiales;f__Lachnospiraceae;g__Blautia | 0.21 | 0.39 | 0.16 | 0.58 | 0.05 | 0.17 |
| k__Bacteria;p__Firmicutes;c__Clostridia;o__Clostridiales;Other;Other | 0.02 | 0.10 | 0.19 | 0.58 | 0.13 | 0.25 |
| k__Bacteria;p__Bacteroidetes;c__Bacteroidia;o__Bacteroidales;f__S24-7;Other | 0.07 | 0.21 | 0.21 | 0.58 | 0.07 | 0.18 |
| k__Bacteria;p__Firmicutes;c__Clostridia;o__Clostridiales;f__Lachnospiraceae;g__Coprococcus | 0.29 | 0.52 | 0.21 | 0.58 | 0.13 | 0.25 |
| k__Bacteria;p__Firmicutes;c__Clostridia;o__Clostridiales;f__Ruminococcaceae;g__Ruminococcus | 0.55 | 0.66 | 0.23 | 0.58 | 0.48 | 0.55 |
| k__Bacteria;p__Verrucomicrobia;c__Verrucomicrobiae;o__Verrucomicrobiales;f__Verrucomicrobiaceae;g__Akkermansia | 0.14 | 0.33 | 0.26 | 0.60 | 0.09 | 0.21 |
| k__Bacteria;p__Actinobacteria;c__Actinobacteria;o__Bifidobacteriales;f__Bifidobacteriaceae;g__Bifidobacterium | 0.69 | 0.76 | 0.28 | 0.60 | 0.85 | 0.94 |
| k__Bacteria;p__Bacteroidetes;c__Bacteroidia;o__Bacteroidales;f__Porphyromonadaceae;g__Parabacteroides | 0.43 | 0.61 | 0.37 | 0.68 | 0.24 | 0.38 |
| k__Bacteria;p__Bacteroidetes;c__Bacteroidia;o__Bacteroidales;f__Bacteroidaceae;g__Bacteroides | 0.46 | 0.62 | 0.39 | 0.68 | 0.30 | 0.41 |
| k__Bacteria;p__Firmicutes;c__Bacilli;o__Bacillales;f__Planococcaceae;Other | 0.06 | 0.21 | 0.40 | 0.68 | 3.14 × 10^−4^ | 3.14 × 10^−3^ |
| k__Bacteria;p__Proteobacteria;c__Gammaproteobacteria;o__Enterobacteriales;f__Enterobacteriaceae;g__Proteus | 0.83 | 0.86 | 0.41 | 0.68 | 0.01 | 0.06 |
| k__Bacteria;p__Proteobacteria;c__Gammaproteobacteria;o__Pseudomonadales;f__Moraxellaceae;g__Acinetobacter | 0.99 | 0.99 | 0.43 | 0.68 | 0.94 | 0.94 |
| k__Bacteria;p__Proteobacteria;c__Betaproteobacteria;o__Burkholderiales;f__Comamonadaceae;Other | 0.63 | 0.73 | 0.59 | 0.85 | 0.92 | 0.94 |
| k__Bacteria;p__Firmicutes;c__Bacilli;o__Bacillales;f__Staphylococcaceae;g__Staphylococcus | 0.36 | 0.57 | 0.60 | 0.85 | 1.11 × 10^−3^ | 8.34 × 10^−3^ |
| k__Bacteria;p__Firmicutes;c__Clostridia;o__Clostridiales;f__Lachnospiraceae;g__[Ruminococcus] | 0.03 | 0.14 | 0.62 | 0.85 | 0.15 | 0.27 |
| Unassigned;Other;Other;Other;Other;Other | 0.54 | 0.66 | 0.67 | 0.87 | 0.37 | 0.45 |
| k__Bacteria;p__Proteobacteria;c__Betaproteobacteria;o__Burkholderiales;f__Alcaligenaceae;g__Sutterella | 0.34 | 0.56 | 0.70 | 0.88 | 0.31 | 0.41 |
| k__Bacteria;p__Firmicutes;c__Clostridia;o__Clostridiales;f__Peptococcaceae;g__rc4-4 | 0.08 | 0.21 | 0.82 | 0.91 | 0.02 | 0.09 |
| k__Bacteria;p__Firmicutes;c__Erysipelotrichi;o__Erysipelotrichales;f__Erysipelotrichaceae;Other | 0.20 | 0.39 | 0.84 | 0.91 | 0.12 | 0.25 |
| k__Bacteria;p__Firmicutes;c__Clostridia;o__Clostridiales;f__Ruminococcaceae;g__Oscillospira | 0.16 | 0.34 | 0.84 | 0.91 | 0.30 | 0.41 |
| k__Bacteria;p__Actinobacteria;c__Coriobacteriia;o__Coriobacteriales;f__Coriobacteriaceae;g__Adlercreutzia | 0.02 | 0.10 | 0.88 | 0.91 | 0.05 | 0.17 |
| k__Bacteria;p__Firmicutes;c__Clostridia;o__Clostridiales;f__Ruminococcaceae;Other | 0.47 | 0.62 | 0.90 | 0.91 | 0.32 | 0.41 |
| k__Bacteria;p__Firmicutes;c__Bacilli;o__Lactobacillales;f__Lactobacillaceae;g__Lactobacillus | 0.40 | 0.59 | 0.91 | 0.91 | 0.13 | 0.25 |

* interaction term of Sugar and Fiber

^†^ denotes FDR adjusted *p* value

## Pairwise Comparison of Sugar N, Fiber Y vs. Sugar N, Fiber N

| Taxon | logFC | logCPM | *p* | *q** |
| --- | --- | --- | --- | --- |
| k__Bacteria;p__Proteobacteria;c__Gammaproteobacteria;o__Pasteurellales;f__Pasteurellaceae;g__Aggregatibacter | 7.05 | 10.50 | 1.78 × 10^−6^ | 5.33 × 10^−5^ |
| k__Bacteria;p__Firmicutes;c__Bacilli;o__Lactobacillales;f__Streptococcaceae;g__Streptococcus | −4.07 | 18.50 | 2.43 × 10^−4^ | 3.64 × 10^−3^ |
| k__Bacteria;p__Proteobacteria;c__Gammaproteobacteria;o__Enterobacteriales;f__Enterobacteriaceae;Other | −3.74 | 10.74 | 1.56 × 10^−3^ | 0.02 |
| k__Bacteria;p__Actinobacteria;c__Actinobacteria;o__Actinomycetales;f__Corynebacteriaceae;g__Corynebacterium | −2.61 | 16.20 | 0.01 | 0.08 |
| k__Bacteria;p__Firmicutes;c__Bacilli;o__Lactobacillales;f__Enterococcaceae;g__Enterococcus | 3.07 | 17.28 | 3.97 × 10^−3^ | 0.03 |
| k__Bacteria;p__Firmicutes;c__Clostridia;o__Clostridiales;f__Lachnospiraceae;Other | −2.20 | 13.77 | 0.04 | 0.18 |
| k__Bacteria;p__Firmicutes;c__Clostridia;o__Clostridiales;f__Lachnospiraceae;g__Dorea | 2.15 | 10.96 | 0.06 | 0.24 |
| k__Bacteria;p__Firmicutes;c__Clostridia;o__Clostridiales;f__Lachnospiraceae;g__Blautia | 1.93 | 8.81 | 0.08 | 0.31 |
| k__Bacteria;p__Firmicutes;c__Clostridia;o__Clostridiales;Other;Other | −1.20 | 14.42 | 0.24 | 0.55 |
| k__Bacteria;p__Bacteroidetes;c__Bacteroidia;o__Bacteroidales;f__S24-7;Other | −1.24 | 15.71 | 0.22 | 0.55 |
| k__Bacteria;p__Firmicutes;c__Clostridia;o__Clostridiales;f__Lachnospiraceae;g__Coprococcus | 1.29 | 11.04 | 0.23 | 0.55 |
| k__Bacteria;p__Firmicutes;c__Clostridia;o__Clostridiales;f__Ruminococcaceae;g__Ruminococcus | 1.46 | 11.54 | 0.17 | 0.51 |
| k__Bacteria;p__Verrucomicrobia;c__Verrucomicrobiae;o__Verrucomicrobiales;f__Verrucomicrobiaceae;g__Akkermansia | 1.67 | 12.30 | 0.11 | 0.38 |
| k__Bacteria;p__Actinobacteria;c__Actinobacteria;o__Bifidobacteriales;f__Bifidobacteriaceae;g__Bifidobacterium | −1.00 | 14.38 | 0.32 | 0.63 |
| k__Bacteria;p__Bacteroidetes;c__Bacteroidia;o__Bacteroidales;f__Porphyromonadaceae;g__Parabacteroides | 0.94 | 13.44 | 0.35 | 0.63 |
| k__Bacteria;p__Bacteroidetes;c__Bacteroidia;o__Bacteroidales;f__Bacteroidaceae;g__Bacteroides | 0.94 | 14.63 | 0.35 | 0.63 |
| k__Bacteria;p__Firmicutes;c__Bacilli;o__Bacillales;f__Planococcaceae;Other | 0.91 | 10.90 | 0.40 | 0.66 |
| k__Bacteria;p__Proteobacteria;c__Gammaproteobacteria;o__Enterobacteriales;f__Enterobacteriaceae;g__Proteus | 1.00 | 10.40 | 0.36 | 0.63 |
| k__Bacteria;p__Proteobacteria;c__Gammaproteobacteria;o__Pseudomonadales;f__Moraxellaceae;g__Acinetobacter | −0.76 | 13.43 | 0.45 | 0.71 |
| k__Bacteria;p__Proteobacteria;c__Betaproteobacteria;o__Burkholderiales;f__Comamonadaceae;Other | −0.52 | 10.54 | 0.62 | 0.85 |
| k__Bacteria;p__Firmicutes;c__Bacilli;o__Bacillales;f__Staphylococcaceae;g__Staphylococcus | 0.51 | 18.15 | 0.61 | 0.85 |
| k__Bacteria;p__Firmicutes;c__Clostridia;o__Clostridiales;f__Lachnospiraceae;g__[Ruminococcus] | −0.58 | 11.81 | 0.58 | 0.85 |
| Unassigned;Other;Other;Other;Other;Other | −0.38 | 14.64 | 0.70 | 0.88 |
| k__Bacteria;p__Proteobacteria;c__Betaproteobacteria;o__Burkholderiales;f__Alcaligenaceae;g__Sutterella | 0.43 | 12.85 | 0.68 | 0.88 |
| k__Bacteria;p__Firmicutes;c__Clostridia;o__Clostridiales;f__Peptococcaceae;g__rc4-4 | −0.25 | 10.73 | 0.81 | 0.92 |
| k__Bacteria;p__Firmicutes;c__Erysipelotrichi;o__Erysipelotrichales;f__Erysipelotrichaceae;Other | −0.24 | 10.13 | 0.83 | 0.92 |
| k__Bacteria;p__Firmicutes;c__Clostridia;o__Clostridiales;f__Ruminococcaceae;g__Oscillospira | 0.20 | 11.35 | 0.85 | 0.92 |
| k__Bacteria;p__Actinobacteria;c__Coriobacteriia;o__Coriobacteriales;f__Coriobacteriaceae;g__Adlercreutzia | −0.14 | 10.59 | 0.90 | 0.92 |
| k__Bacteria;p__Firmicutes;c__Clostridia;o__Clostridiales;f__Ruminococcaceae;Other | 0.11 | 15.86 | 0.91 | 0.92 |
| k__Bacteria;p__Firmicutes;c__Bacilli;o__Lactobacillales;f__Lactobacillaceae;g__Lactobacillus | 0.10 | 17.50 | 0.92 | 0.92 |

* denotes FDR adjusted *p* value

## Pairwise Comparison of Sugar Y, Fiber N vs. Sugar N, Fiber N

| Taxon | logFC | logCPM | *p* | *q** |
| --- | --- | --- | --- | --- |
| k__Bacteria;p__Proteobacteria;c__Gammaproteobacteria;o__Pasteurellales;f__Pasteurellaceae;g__Aggregatibacter | 10.93 | 13.72 | 5.57 × 10^−6^ | 1.67 × 10^−4^ |
| k__Bacteria;p__Firmicutes;c__Bacilli;o__Lactobacillales;f__Streptococcaceae;g__Streptococcus | 1.02 | 18.59 | 0.37 | 0.46 |
| k__Bacteria;p__Proteobacteria;c__Gammaproteobacteria;o__Enterobacteriales;f__Enterobacteriaceae;Other | −2.91 | 10.58 | 0.02 | 0.16 |
| k__Bacteria;p__Actinobacteria;c__Actinobacteria;o__Actinomycetales;f__Corynebacteriaceae;g__Corynebacterium | −1.49 | 15.96 | 0.15 | 0.35 |
| k__Bacteria;p__Firmicutes;c__Bacilli;o__Lactobacillales;f__Enterococcaceae;g__Enterococcus | 3.39 | 16.53 | 8.60 × 10^−3^ | 0.13 |
| k__Bacteria;p__Firmicutes;c__Clostridia;o__Clostridiales;f__Lachnospiraceae;Other | −2.39 | 12.99 | 0.04 | 0.23 |
| k__Bacteria;p__Firmicutes;c__Clostridia;o__Clostridiales;f__Lachnospiraceae;g__Dorea | −2.21 | 8.38 | 0.10 | 0.29 |
| k__Bacteria;p__Firmicutes;c__Clostridia;o__Clostridiales;f__Lachnospiraceae;g__Blautia | −1.80 | 7.09 | 0.21 | 0.45 |
| k__Bacteria;p__Firmicutes;c__Clostridia;o__Clostridiales;Other;Other | −2.34 | 13.54 | 0.04 | 0.23 |
| k__Bacteria;p__Bacteroidetes;c__Bacteroidia;o__Bacteroidales;f__S24-7;Other | −2.11 | 14.90 | 0.06 | 0.24 |
| k__Bacteria;p__Firmicutes;c__Clostridia;o__Clostridiales;f__Lachnospiraceae;g__Coprococcus | −1.25 | 9.54 | 0.32 | 0.46 |
| k__Bacteria;p__Firmicutes;c__Clostridia;o__Clostridiales;f__Ruminococcaceae;g__Ruminococcus | −0.24 | 10.63 | 0.87 | 0.87 |
| k__Bacteria;p__Verrucomicrobia;c__Verrucomicrobiae;o__Verrucomicrobiales;f__Verrucomicrobiaceae;g__Akkermansia | −2.24 | 9.91 | 0.23 | 0.46 |
| k__Bacteria;p__Actinobacteria;c__Actinobacteria;o__Bifidobacteriales;f__Bifidobacteriaceae;g__Bifidobacterium | −0.91 | 14.27 | 0.38 | 0.46 |
| k__Bacteria;p__Bacteroidetes;c__Bacteroidia;o__Bacteroidales;f__Porphyromonadaceae;g__Parabacteroides | −0.83 | 11.79 | 0.47 | 0.54 |
| k__Bacteria;p__Bacteroidetes;c__Bacteroidia;o__Bacteroidales;f__Bacteroidaceae;g__Bacteroides | −0.79 | 13.00 | 0.50 | 0.55 |
| k__Bacteria;p__Firmicutes;c__Bacilli;o__Bacillales;f__Planococcaceae;Other | 2.18 | 11.98 | 0.09 | 0.27 |
| k__Bacteria;p__Proteobacteria;c__Gammaproteobacteria;o__Enterobacteriales;f__Enterobacteriaceae;g__Proteus | 0.40 | 9.87 | 0.74 | 0.80 |
| k__Bacteria;p__Proteobacteria;c__Gammaproteobacteria;o__Pseudomonadales;f__Moraxellaceae;g__Acinetobacter | 0.30 | 13.31 | 0.78 | 0.81 |
| k__Bacteria;p__Proteobacteria;c__Betaproteobacteria;o__Burkholderiales;f__Comamonadaceae;Other | 1.02 | 10.78 | 0.35 | 0.46 |
| k__Bacteria;p__Firmicutes;c__Bacilli;o__Bacillales;f__Staphylococcaceae;g__Staphylococcus | 1.11 | 18.13 | 0.29 | 0.46 |
| k__Bacteria;p__Firmicutes;c__Clostridia;o__Clostridiales;f__Lachnospiraceae;g__[Ruminococcus] | −3.00 | 10.74 | 0.05 | 0.23 |
| Unassigned;Other;Other;Other;Other;Other | 0.93 | 14.64 | 0.32 | 0.46 |
| k__Bacteria;p__Proteobacteria;c__Betaproteobacteria;o__Burkholderiales;f__Alcaligenaceae;g__Sutterella | −1.28 | 11.59 | 0.33 | 0.46 |
| k__Bacteria;p__Firmicutes;c__Clostridia;o__Clostridiales;f__Peptococcaceae;g__rc4-4 | −2.66 | 10.26 | 0.08 | 0.26 |
| k__Bacteria;p__Firmicutes;c__Erysipelotrichi;o__Erysipelotrichales;f__Erysipelotrichaceae;Other | −1.64 | 9.29 | 0.24 | 0.46 |
| k__Bacteria;p__Firmicutes;c__Clostridia;o__Clostridiales;f__Ruminococcaceae;g__Oscillospira | −1.20 | 10.29 | 0.36 | 0.46 |
| k__Bacteria;p__Actinobacteria;c__Coriobacteriia;o__Coriobacteriales;f__Coriobacteriaceae;g__Adlercreutzia | −2.46 | 9.51 | 0.05 | 0.23 |
| k__Bacteria;p__Firmicutes;c__Clostridia;o__Clostridiales;f__Ruminococcaceae;Other | −1.14 | 15.01 | 0.27 | 0.46 |
| k__Bacteria;p__Firmicutes;c__Bacilli;o__Lactobacillales;f__Lactobacillaceae;g__Lactobacillus | 1.30 | 17.31 | 0.15 | 0.35 |

* denotes FDR adjusted *p* value

## Pairwise Comparison of Sugar Y, Fiber Y vs. Sugar N, Fiber Y

| Taxon | logFC | logCPM | *p* | *q** |
| --- | --- | --- | --- | --- |
| k__Bacteria;p__Proteobacteria;c__Gammaproteobacteria;o__Pasteurellales;f__Pasteurellaceae;g__Aggregatibacter | 7.18 | 18.53 | 2.74 × 10^−3^ | 0.02 |
| k__Bacteria;p__Firmicutes;c__Bacilli;o__Lactobacillales;f__Streptococcaceae;g__Streptococcus | 3.13 | 17.89 | 0.02 | 0.07 |
| k__Bacteria;p__Proteobacteria;c__Gammaproteobacteria;o__Enterobacteriales;f__Enterobacteriaceae;Other | 6.25 | 13.61 | 2.22 × 10^−4^ | 6.65 × 10^−3^ |
| k__Bacteria;p__Actinobacteria;c__Actinobacteria;o__Actinomycetales;f__Corynebacteriaceae;g__Corynebacterium | −0.89 | 13.81 | 0.35 | 0.71 |
| k__Bacteria;p__Firmicutes;c__Bacilli;o__Lactobacillales;f__Enterococcaceae;g__Enterococcus | −4.45 | 17.66 | 2.57 × 10^−3^ | 0.02 |
| k__Bacteria;p__Firmicutes;c__Clostridia;o__Clostridiales;f__Lachnospiraceae;Other | 0.72 | 13.16 | 0.41 | 0.72 |
| k__Bacteria;p__Firmicutes;c__Clostridia;o__Clostridiales;f__Lachnospiraceae;g__Dorea | −1.09 | 11.70 | 0.24 | 0.59 |
| k__Bacteria;p__Firmicutes;c__Clostridia;o__Clostridiales;f__Lachnospiraceae;g__Blautia | 2.04 | 10.64 | 0.14 | 0.41 |
| k__Bacteria;p__Firmicutes;c__Clostridia;o__Clostridiales;Other;Other | −0.56 | 13.90 | 0.48 | 0.72 |
| k__Bacteria;p__Bacteroidetes;c__Bacteroidia;o__Bacteroidales;f__S24-7;Other | 0.44 | 15.41 | 0.59 | 0.73 |
| k__Bacteria;p__Firmicutes;c__Clostridia;o__Clostridiales;f__Lachnospiraceae;g__Coprococcus | 1.61 | 12.88 | 0.13 | 0.41 |
| k__Bacteria;p__Firmicutes;c__Clostridia;o__Clostridiales;f__Ruminococcaceae;g__Ruminococcus | −0.78 | 11.87 | 0.35 | 0.71 |
| k__Bacteria;p__Verrucomicrobia;c__Verrucomicrobiae;o__Verrucomicrobiales;f__Verrucomicrobiaceae;g__Akkermansia | 0.67 | 13.71 | 0.55 | 0.73 |
| k__Bacteria;p__Actinobacteria;c__Actinobacteria;o__Bifidobacteriales;f__Bifidobacteriaceae;g__Bifidobacterium | 0.18 | 13.81 | 0.84 | 0.87 |
| k__Bacteria;p__Bacteroidetes;c__Bacteroidia;o__Bacteroidales;f__Porphyromonadaceae;g__Parabacteroides | 0.65 | 14.06 | 0.47 | 0.72 |
| k__Bacteria;p__Bacteroidetes;c__Bacteroidia;o__Bacteroidales;f__Bacteroidaceae;g__Bacteroides | 0.74 | 15.10 | 0.43 | 0.72 |
| k__Bacteria;p__Firmicutes;c__Bacilli;o__Bacillales;f__Planococcaceae;Other | −4.15 | 10.34 | 1.62 × 10^−3^ | 0.02 |
| k__Bacteria;p__Proteobacteria;c__Gammaproteobacteria;o__Enterobacteriales;f__Enterobacteriaceae;g__Proteus | 5.80 | 15.89 | 1.32 × 10^−3^ | 0.02 |
| k__Bacteria;p__Proteobacteria;c__Gammaproteobacteria;o__Pseudomonadales;f__Moraxellaceae;g__Acinetobacter | 0.42 | 13.39 | 0.68 | 0.73 |
| k__Bacteria;p__Proteobacteria;c__Betaproteobacteria;o__Burkholderiales;f__Comamonadaceae;Other | 1.16 | 10.68 | 0.25 | 0.59 |
| k__Bacteria;p__Firmicutes;c__Bacilli;o__Bacillales;f__Staphylococcaceae;g__Staphylococcus | −3.20 | 17.44 | 4.23 × 10^−3^ | 0.02 |
| k__Bacteria;p__Firmicutes;c__Clostridia;o__Clostridiales;f__Lachnospiraceae;g__[Ruminococcus] | −0.38 | 11.64 | 0.67 | 0.73 |
| Unassigned;Other;Other;Other;Other;Other | 1.98 | 15.92 | 0.06 | 0.23 |
| k__Bacteria;p__Proteobacteria;c__Betaproteobacteria;o__Burkholderiales;f__Alcaligenaceae;g__Sutterella | 0.44 | 13.23 | 0.60 | 0.73 |
| k__Bacteria;p__Firmicutes;c__Clostridia;o__Clostridiales;f__Peptococcaceae;g__rc4-4 | 1.15 | 11.63 | 0.23 | 0.59 |
| k__Bacteria;p__Firmicutes;c__Erysipelotrichi;o__Erysipelotrichales;f__Erysipelotrichaceae;Other | 0.47 | 10.65 | 0.62 | 0.73 |
| k__Bacteria;p__Firmicutes;c__Clostridia;o__Clostridiales;f__Ruminococcaceae;g__Oscillospira | −0.47 | 11.64 | 0.58 | 0.73 |
| k__Bacteria;p__Actinobacteria;c__Coriobacteriia;o__Coriobacteriales;f__Coriobacteriaceae;g__Adlercreutzia | −0.11 | 10.94 | 0.89 | 0.89 |
| k__Bacteria;p__Firmicutes;c__Clostridia;o__Clostridiales;f__Ruminococcaceae;Other | 0.40 | 16.19 | 0.63 | 0.73 |
| k__Bacteria;p__Firmicutes;c__Bacilli;o__Lactobacillales;f__Lactobacillaceae;g__Lactobacillus | −0.85 | 17.21 | 0.41 | 0.72 |

* denotes FDR adjusted *p* value

## Pairwise Comparison of Sugar Y, Fiber Y vs. Sugar Y, Fiber N

| Observation | logFC | logCPM | *p* | *q** |
| --- | --- | --- | --- | --- |
| k__Bacteria;p__Proteobacteria;c__Gammaproteobacteria;o__Pasteurellales;f__Pasteurellaceae;g__Aggregatibacter | 5.17 | 17.49 | 0.02 | 0.04 |
| k__Bacteria;p__Firmicutes;c__Bacilli;o__Lactobacillales;f__Streptococcaceae;g__Streptococcus | −0.69 | 18.16 | 0.57 | 0.64 |
| k__Bacteria;p__Proteobacteria;c__Gammaproteobacteria;o__Enterobacteriales;f__Enterobacteriaceae;Other | 5.33 | 12.91 | 1.25 × 10^−3^ | 5.34 × 10^−3^ |
| k__Bacteria;p__Actinobacteria;c__Actinobacteria;o__Actinomycetales;f__Corynebacteriaceae;g__Corynebacterium | −2.33 | 14.13 | 0.02 | 0.04 |
| k__Bacteria;p__Firmicutes;c__Bacilli;o__Lactobacillales;f__Enterococcaceae;g__Enterococcus | −4.33 | 16.81 | 3.39 × 10^−3^ | 0.01 |
| k__Bacteria;p__Firmicutes;c__Clostridia;o__Clostridiales;f__Lachnospiraceae;Other | 1.84 | 12.67 | 0.06 | 0.11 |
| k__Bacteria;p__Firmicutes;c__Clostridia;o__Clostridiales;f__Lachnospiraceae;g__Dorea | 4.29 | 10.14 | 8.11 × 10^−4^ | 4.87 × 10^−3^ |
| k__Bacteria;p__Firmicutes;c__Clostridia;o__Clostridiales;f__Lachnospiraceae;g__Blautia | 6.29 | 10.49 | 2.24 × 10^−4^ | 3.36 × 10^−3^ |
| k__Bacteria;p__Firmicutes;c__Clostridia;o__Clostridiales;Other;Other | 1.14 | 12.85 | 0.25 | 0.31 |
| k__Bacteria;p__Bacteroidetes;c__Bacteroidia;o__Bacteroidales;f__S24-7;Other | 1.90 | 14.89 | 0.09 | 0.12 |
| k__Bacteria;p__Firmicutes;c__Clostridia;o__Clostridiales;f__Lachnospiraceae;g__Coprococcus | 4.28 | 11.98 | 1.70 × 10^−3^ | 6.37 × 10^−3^ |
| k__Bacteria;p__Firmicutes;c__Clostridia;o__Clostridiales;f__Ruminococcaceae;g__Ruminococcus | 0.91 | 11.13 | 0.52 | 0.61 |
| k__Bacteria;p__Verrucomicrobia;c__Verrucomicrobiae;o__Verrucomicrobiales;f__Verrucomicrobiaceae;g__Akkermansia | 5.93 | 13.15 | 1.05 × 10^−3^ | 5.23 × 10^−3^ |
| k__Bacteria;p__Actinobacteria;c__Actinobacteria;o__Bifidobacteriales;f__Bifidobacteriaceae;g__Bifidobacterium | −0.41 | 13.75 | 0.69 | 0.74 |
| k__Bacteria;p__Bacteroidetes;c__Bacteroidia;o__Bacteroidales;f__Porphyromonadaceae;g__Parabacteroides | 3.12 | 13.68 | 8.25 × 10^−3^ | 0.02 |
| k__Bacteria;p__Bacteroidetes;c__Bacteroidia;o__Bacteroidales;f__Bacteroidaceae;g__Bacteroides | 2.85 | 14.77 | 0.02 | 0.04 |
| k__Bacteria;p__Firmicutes;c__Bacilli;o__Bacillales;f__Planococcaceae;Other | −5.50 | 11.81 | 1.07 × 10^−4^ | 3.21 × 10^−3^ |
| k__Bacteria;p__Proteobacteria;c__Gammaproteobacteria;o__Enterobacteriales;f__Enterobacteriaceae;g__Proteus | 6.24 | 15.14 | 6.67 × 10^−4^ | 4.87 × 10^−3^ |
| k__Bacteria;p__Proteobacteria;c__Gammaproteobacteria;o__Pseudomonadales;f__Moraxellaceae;g__Acinetobacter | −0.06 | 12.85 | 0.96 | 0.96 |
| k__Bacteria;p__Proteobacteria;c__Betaproteobacteria;o__Burkholderiales;f__Comamonadaceae;Other | −0.30 | 10.74 | 0.78 | 0.81 |
| k__Bacteria;p__Firmicutes;c__Bacilli;o__Bacillales;f__Staphylococcaceae;g__Staphylococcus | −4.33 | 17.70 | 3.66 × 10^−4^ | 3.66 × 10^−3^ |
| k__Bacteria;p__Firmicutes;c__Clostridia;o__Clostridiales;f__Lachnospiraceae;g__[Ruminococcus] | 2.55 | 10.54 | 0.05 | 0.09 |
| Unassigned;Other;Other;Other;Other;Other | 0.84 | 15.39 | 0.41 | 0.50 |
| k__Bacteria;p__Proteobacteria;c__Betaproteobacteria;o__Burkholderiales;f__Alcaligenaceae;g__Sutterella | 2.39 | 12.73 | 0.08 | 0.12 |
| k__Bacteria;p__Firmicutes;c__Clostridia;o__Clostridiales;f__Peptococcaceae;g__rc4-4 | 3.67 | 11.26 | 4.13 × 10^−3^ | 0.01 |
| k__Bacteria;p__Firmicutes;c__Erysipelotrichi;o__Erysipelotrichales;f__Erysipelotrichaceae;Other | 2.59 | 10.25 | 0.08 | 0.12 |
| k__Bacteria;p__Firmicutes;c__Clostridia;o__Clostridiales;f__Ruminococcaceae;g__Oscillospira | 2.13 | 10.75 | 0.06 | 0.11 |
| k__Bacteria;p__Actinobacteria;c__Coriobacteriia;o__Coriobacteriales;f__Coriobacteriaceae;g__Adlercreutzia | 3.05 | 10.04 | 0.01 | 0.03 |
| k__Bacteria;p__Firmicutes;c__Clostridia;o__Clostridiales;f__Ruminococcaceae;Other | 1.86 | 15.72 | 0.08 | 0.12 |
| k__Bacteria;p__Firmicutes;c__Bacilli;o__Lactobacillales;f__Lactobacillaceae;g__Lactobacillus | −1.56 | 17.17 | 0.14 | 0.18 |

* denotes FDR adjusted *p* value

# Differential Analysis at Family Level

## Omnibus Tests

| Taxon | Sugar | | Fiber | | Sugar::Fiber* | |
| --- | --- | --- | --- | --- | --- | --- |
|  | *p* | *q*^†^ | *p* | *q*^†^ | *p* | *q*^†^ |
| k__Bacteria;p__Firmicutes;c__Bacilli;o__Lactobacillales;f__Enterococcaceae | 4.50 × 10^−3^ | 0.05 | 2.85 × 10^−4^ | 6.55 × 10^−3^ | 5.64 × 10^−7^ | 1.30 × 10^−5^ |
| k__Bacteria;p__Firmicutes;c__Bacilli;o__Bacillales;f__Planococcaceae | 0.07 | 0.27 | 0.06 | 0.27 | 1.40 × 10^−5^ | 1.61 × 10^−4^ |
| k__Bacteria;p__Firmicutes;c__Bacilli;o__Bacillales;f__Staphylococcaceae | 0.36 | 0.59 | 0.23 | 0.49 | 7.41 × 10^−5^ | 5.68 × 10^−4^ |
| k__Bacteria;p__Proteobacteria;c__Gammaproteobacteria;o__Enterobacteriales;f__Enterobacteriaceae | 0.41 | 0.59 | 0.85 | 0.93 | 3.56 × 10^−3^ | 0.02 |
| k__Bacteria;p__Firmicutes;c__Bacilli;o__Lactobacillales;f__Lactobacillaceae | 0.18 | 0.38 | 0.08 | 0.29 | 0.01 | 0.05 |
| k__Bacteria;p__Firmicutes;c__Clostridia;o__Clostridiales;f__Peptococcaceae | 0.11 | 0.29 | 0.60 | 0.81 | 0.04 | 0.16 |
| k__Bacteria;p__Firmicutes;c__Clostridia;o__Clostridiales;f__Lachnospiraceae | 0.05 | 0.27 | 0.51 | 0.74 | 0.06 | 0.16 |
| k__Bacteria;p__Actinobacteria;c__Coriobacteriia;o__Coriobacteriales;f__Coriobacteriaceae | 0.02 | 0.15 | 0.94 | 0.94 | 0.06 | 0.16 |
| k__Bacteria;p__Firmicutes;c__Erysipelotrichi;o__Erysipelotrichales;f__Erysipelotrichaceae | 0.11 | 0.29 | 0.67 | 0.83 | 0.08 | 0.20 |
| k__Bacteria;p__Verrucomicrobia;c__Verrucomicrobiae;o__Verrucomicrobiales;f__Verrucomicrobiaceae | 0.23 | 0.45 | 0.14 | 0.43 | 0.15 | 0.33 |
| k__Bacteria;p__Bacteroidetes;c__Bacteroidia;o__Bacteroidales;f__S24-7 | 0.13 | 0.29 | 0.51 | 0.74 | 0.16 | 0.33 |
| k__Bacteria;p__Firmicutes;c__Bacilli;o__Lactobacillales;f__Streptococcaceae | 0.80 | 0.88 | 0.02 | 0.15 | 0.22 | 0.42 |
| k__Bacteria;p__Firmicutes;c__Clostridia;o__Clostridiales;Other | 0.07 | 0.27 | 0.35 | 0.62 | 0.32 | 0.57 |
| k__Bacteria;p__Proteobacteria;c__Betaproteobacteria;o__Burkholderiales;f__Comamonadaceae | 0.41 | 0.59 | 0.91 | 0.94 | 0.49 | 0.68 |
| k__Bacteria;p__Firmicutes;c__Clostridia;o__Clostridiales;f__Ruminococcaceae | 0.58 | 0.78 | 0.72 | 0.83 | 0.51 | 0.68 |
| k__Bacteria;p__Actinobacteria;c__Actinobacteria;o__Actinomycetales;f__Corynebacteriaceae | 0.12 | 0.29 | 0.03 | 0.15 | 0.53 | 0.68 |
| k__Bacteria;p__Proteobacteria;c__Gammaproteobacteria;o__Pseudomonadales;f__Moraxellaceae | 0.72 | 0.83 | 0.72 | 0.83 | 0.55 | 0.68 |
| Unassigned;Other;Other;Other;Other | 0.37 | 0.59 | 0.27 | 0.51 | 0.57 | 0.68 |
| k__Bacteria;p__Bacteroidetes;c__Bacteroidia;o__Bacteroidales;f__Porphyromonadaceae | 0.84 | 0.88 | 0.15 | 0.43 | 0.61 | 0.68 |
| k__Bacteria;p__Bacteroidetes;c__Bacteroidia;o__Bacteroidales;f__Bacteroidaceae | 0.94 | 0.94 | 0.22 | 0.49 | 0.64 | 0.68 |
| k__Bacteria;p__Proteobacteria;c__Gammaproteobacteria;o__Pasteurellales;f__Pasteurellaceae | 1.30 × 10^−3^ | 0.03 | 0.02 | 0.15 | 0.64 | 0.68 |
| k__Bacteria;p__Proteobacteria;c__Betaproteobacteria;o__Burkholderiales;f__Alcaligenaceae | 0.63 | 0.81 | 0.41 | 0.68 | 0.65 | 0.68 |
| k__Bacteria;p__Actinobacteria;c__Actinobacteria;o__Bifidobacteriales;f__Bifidobacteriaceae | 0.71 | 0.83 | 0.23 | 0.49 | 0.77 | 0.77 |

* interaction term of Sugar and Fiber

^†^ denotes FDR adjusted *p* value

## Pairwise Comparison of Sugar N, Fiber Y vs. Sugar N, Fiber N

| Taxon | logFC | logCPM | *p* | *q** |
| --- | --- | --- | --- | --- |
| k__Bacteria;p__Firmicutes;c__Bacilli;o__Lactobacillales;f__Enterococcaceae | 3.44 | 17.24 | 2.02 × 10^−3^ | 0.01 |
| k__Bacteria;p__Firmicutes;c__Bacilli;o__Bacillales;f__Planococcaceae | 0.93 | 10.87 | 0.38 | 0.67 |
| k__Bacteria;p__Firmicutes;c__Bacilli;o__Bacillales;f__Staphylococcaceae | −0.22 | 18.28 | 0.81 | 0.89 |
| k__Bacteria;p__Proteobacteria;c__Gammaproteobacteria;o__Enterobacteriales;f__Enterobacteriaceae | −1.61 | 11.84 | 0.09 | 0.36 |
| k__Bacteria;p__Firmicutes;c__Bacilli;o__Lactobacillales;f__Lactobacillaceae | −0.02 | 17.29 | 0.98 | 0.98 |
| k__Bacteria;p__Firmicutes;c__Clostridia;o__Clostridiales;f__Peptococcaceae | 0.03 | 10.81 | 0.98 | 0.98 |
| k__Bacteria;p__Firmicutes;c__Clostridia;o__Clostridiales;f__Lachnospiraceae | −0.34 | 14.22 | 0.70 | 0.89 |
| k__Bacteria;p__Actinobacteria;c__Coriobacteriia;o__Coriobacteriales;f__Coriobacteriaceae | 0.24 | 10.69 | 0.80 | 0.89 |
| k__Bacteria;p__Firmicutes;c__Erysipelotrichi;o__Erysipelotrichales;f__Erysipelotrichaceae | −0.53 | 10.80 | 0.57 | 0.77 |
| k__Bacteria;p__Verrucomicrobia;c__Verrucomicrobiae;o__Verrucomicrobiales;f__Verrucomicrobiaceae | 2.17 | 12.46 | 0.07 | 0.34 |
| k__Bacteria;p__Bacteroidetes;c__Bacteroidia;o__Bacteroidales;f__S24-7 | −0.92 | 15.47 | 0.28 | 0.65 |
| k__Bacteria;p__Firmicutes;c__Bacilli;o__Lactobacillales;f__Streptococcaceae | −3.88 | 18.30 | 3.89 × 10^−4^ | 8.94 × 10^−3^ |
| k__Bacteria;p__Firmicutes;c__Clostridia;o__Clostridiales;Other | −0.55 | 14.27 | 0.53 | 0.77 |
| k__Bacteria;p__Proteobacteria;c__Betaproteobacteria;o__Burkholderiales;f__Comamonadaceae | −0.95 | 10.74 | 0.34 | 0.65 |
| k__Bacteria;p__Firmicutes;c__Clostridia;o__Clostridiales;f__Ruminococcaceae | 0.26 | 15.83 | 0.76 | 0.89 |
| k__Bacteria;p__Actinobacteria;c__Actinobacteria;o__Actinomycetales;f__Corynebacteriaceae | −3.32 | 16.56 | 1.06 × 10^−3^ | 0.01 |
| k__Bacteria;p__Proteobacteria;c__Gammaproteobacteria;o__Pseudomonadales;f__Moraxellaceae | −1.17 | 13.62 | 0.21 | 0.60 |
| Unassigned;Other;Other;Other;Other | −0.68 | 14.73 | 0.42 | 0.69 |
| k__Bacteria;p__Bacteroidetes;c__Bacteroidia;o__Bacteroidales;f__Porphyromonadaceae | 1.02 | 13.15 | 0.29 | 0.65 |
| k__Bacteria;p__Bacteroidetes;c__Bacteroidia;o__Bacteroidales;f__Bacteroidaceae | 0.89 | 14.22 | 0.34 | 0.65 |
| k__Bacteria;p__Proteobacteria;c__Gammaproteobacteria;o__Pasteurellales;f__Pasteurellaceae | 4.69 | 11.22 | 1.31 × 10^−3^ | 0.01 |
| k__Bacteria;p__Proteobacteria;c__Betaproteobacteria;o__Burkholderiales;f__Alcaligenaceae | 0.57 | 12.57 | 0.54 | 0.77 |
| k__Bacteria;p__Actinobacteria;c__Actinobacteria;o__Bifidobacteriales;f__Bifidobacteriaceae | −1.10 | 14.27 | 0.20 | 0.60 |

* denotes FDR adjusted *p* value

## Pairwise Comparison of Sugar Y, Fiber N vs. Sugar N, Fiber N

| Taxon | logFC | logCPM | *p* | *q** |
| --- | --- | --- | --- | --- |
| k__Bacteria;p__Firmicutes;c__Bacilli;o__Lactobacillales;f__Enterococcaceae | 4.05 | 17.34 | 3.36 × 10^−3^ | 0.04 |
| k__Bacteria;p__Firmicutes;c__Bacilli;o__Bacillales;f__Planococcaceae | 2.36 | 12.43 | 0.08 | 0.43 |
| k__Bacteria;p__Firmicutes;c__Bacilli;o__Bacillales;f__Staphylococcaceae | 0.93 | 18.36 | 0.38 | 0.59 |
| k__Bacteria;p__Proteobacteria;c__Gammaproteobacteria;o__Enterobacteriales;f__Enterobacteriaceae | −0.99 | 11.25 | 0.36 | 0.59 |
| k__Bacteria;p__Firmicutes;c__Bacilli;o__Lactobacillales;f__Lactobacillaceae | 1.25 | 17.37 | 0.15 | 0.43 |
| k__Bacteria;p__Firmicutes;c__Clostridia;o__Clostridiales;f__Peptococcaceae | −1.98 | 10.33 | 0.21 | 0.44 |
| k__Bacteria;p__Firmicutes;c__Clostridia;o__Clostridiales;f__Lachnospiraceae | −1.84 | 13.53 | 0.10 | 0.43 |
| k__Bacteria;p__Actinobacteria;c__Coriobacteriia;o__Coriobacteriales;f__Coriobacteriaceae | −2.40 | 9.66 | 0.07 | 0.43 |
| k__Bacteria;p__Firmicutes;c__Erysipelotrichi;o__Erysipelotrichales;f__Erysipelotrichaceae | −1.78 | 10.08 | 0.20 | 0.44 |
| k__Bacteria;p__Verrucomicrobia;c__Verrucomicrobiae;o__Verrucomicrobiales;f__Verrucomicrobiaceae | −1.73 | 10.07 | 0.36 | 0.59 |
| k__Bacteria;p__Bacteroidetes;c__Bacteroidia;o__Bacteroidales;f__S24-7 | −1.54 | 15.12 | 0.18 | 0.44 |
| k__Bacteria;p__Firmicutes;c__Bacilli;o__Lactobacillales;f__Streptococcaceae | 0.26 | 18.09 | 0.80 | 0.88 |
| k__Bacteria;p__Firmicutes;c__Clostridia;o__Clostridiales;Other | −1.68 | 13.71 | 0.14 | 0.43 |
| k__Bacteria;p__Proteobacteria;c__Betaproteobacteria;o__Burkholderiales;f__Comamonadaceae | 0.77 | 10.82 | 0.49 | 0.70 |
| k__Bacteria;p__Firmicutes;c__Clostridia;o__Clostridiales;f__Ruminococcaceae | −0.50 | 15.37 | 0.63 | 0.85 |
| k__Bacteria;p__Actinobacteria;c__Actinobacteria;o__Actinomycetales;f__Corynebacteriaceae | −1.41 | 15.78 | 0.15 | 0.43 |
| k__Bacteria;p__Proteobacteria;c__Gammaproteobacteria;o__Pseudomonadales;f__Moraxellaceae | −0.34 | 13.13 | 0.74 | 0.85 |
| Unassigned;Other;Other;Other;Other | 0.80 | 14.68 | 0.38 | 0.59 |
| k__Bacteria;p__Bacteroidetes;c__Bacteroidia;o__Bacteroidales;f__Porphyromonadaceae | −0.20 | 12.06 | 0.87 | 0.91 |
| k__Bacteria;p__Bacteroidetes;c__Bacteroidia;o__Bacteroidales;f__Bacteroidaceae | −0.08 | 13.33 | 0.95 | 0.95 |
| k__Bacteria;p__Proteobacteria;c__Gammaproteobacteria;o__Pasteurellales;f__Pasteurellaceae | 6.03 | 12.11 | 1.31 × 10^−3^ | 0.03 |
| k__Bacteria;p__Proteobacteria;c__Betaproteobacteria;o__Burkholderiales;f__Alcaligenaceae | −0.53 | 11.85 | 0.70 | 0.85 |
| k__Bacteria;p__Actinobacteria;c__Actinobacteria;o__Bifidobacteriales;f__Bifidobacteriaceae | −0.35 | 14.54 | 0.74 | 0.85 |

* denotes FDR adjusted *p* value

## Pairwise Comparison of Sugar Y, Fiber Y vs. Sugar N, Fiber Y

| Taxon | logFC | logCPM | *p* | *q** |
| --- | --- | --- | --- | --- |
| k__Bacteria;p__Firmicutes;c__Bacilli;o__Lactobacillales;f__Enterococcaceae | −5.82 | 18.20 | 1.27 × 10^−4^ | 9.71 × 10^−4^ |
| k__Bacteria;p__Firmicutes;c__Bacilli;o__Bacillales;f__Planococcaceae | −5.51 | 10.60 | 2.11 × 10^−5^ | 2.43 × 10^−4^ |
| k__Bacteria;p__Firmicutes;c__Bacilli;o__Bacillales;f__Staphylococcaceae | −4.98 | 18.12 | 1.08 × 10^−5^ | 2.43 × 10^−4^ |
| k__Bacteria;p__Proteobacteria;c__Gammaproteobacteria;o__Enterobacteriales;f__Enterobacteriaceae | 4.74 | 15.53 | 2.94 × 10^−3^ | 0.01 |
| k__Bacteria;p__Firmicutes;c__Bacilli;o__Lactobacillales;f__Lactobacillaceae | −1.91 | 17.40 | 0.06 | 0.19 |
| k__Bacteria;p__Firmicutes;c__Clostridia;o__Clostridiales;f__Peptococcaceae | 1.36 | 11.67 | 0.14 | 0.35 |
| k__Bacteria;p__Firmicutes;c__Clostridia;o__Clostridiales;f__Lachnospiraceae | 0.32 | 14.48 | 0.70 | 0.74 |
| k__Bacteria;p__Actinobacteria;c__Coriobacteriia;o__Coriobacteriales;f__Coriobacteriaceae | −0.07 | 10.95 | 0.94 | 0.94 |
| k__Bacteria;p__Firmicutes;c__Erysipelotrichi;o__Erysipelotrichales;f__Erysipelotrichaceae | 0.79 | 11.20 | 0.39 | 0.67 |
| k__Bacteria;p__Verrucomicrobia;c__Verrucomicrobiae;o__Verrucomicrobiales;f__Verrucomicrobiaceae | 0.84 | 13.79 | 0.45 | 0.69 |
| k__Bacteria;p__Bacteroidetes;c__Bacteroidia;o__Bacteroidales;f__S24-7 | 0.40 | 15.43 | 0.61 | 0.74 |
| k__Bacteria;p__Firmicutes;c__Bacilli;o__Lactobacillales;f__Streptococcaceae | 1.89 | 16.83 | 0.08 | 0.23 |
| k__Bacteria;p__Firmicutes;c__Clostridia;o__Clostridiales;Other | −0.88 | 13.84 | 0.23 | 0.53 |
| k__Bacteria;p__Proteobacteria;c__Betaproteobacteria;o__Burkholderiales;f__Comamonadaceae | −0.42 | 10.56 | 0.65 | 0.74 |
| k__Bacteria;p__Firmicutes;c__Clostridia;o__Clostridiales;f__Ruminococcaceae | 0.31 | 16.37 | 0.70 | 0.74 |
| k__Bacteria;p__Actinobacteria;c__Actinobacteria;o__Actinomycetales;f__Corynebacteriaceae | −2.41 | 13.97 | 6.73 × 10^−3^ | 0.03 |
| k__Bacteria;p__Proteobacteria;c__Gammaproteobacteria;o__Pseudomonadales;f__Moraxellaceae | −0.92 | 13.10 | 0.31 | 0.65 |
| Unassigned;Other;Other;Other;Other | 0.40 | 15.30 | 0.66 | 0.74 |
| k__Bacteria;p__Bacteroidetes;c__Bacteroidia;o__Bacteroidales;f__Porphyromonadaceae | 0.76 | 14.20 | 0.41 | 0.67 |
| k__Bacteria;p__Bacteroidetes;c__Bacteroidia;o__Bacteroidales;f__Bacteroidaceae | 0.80 | 15.24 | 0.41 | 0.67 |
| k__Bacteria;p__Proteobacteria;c__Gammaproteobacteria;o__Pasteurellales;f__Pasteurellaceae | 6.19 | 17.03 | 2.20 × 10^−3^ | 0.01 |
| k__Bacteria;p__Proteobacteria;c__Betaproteobacteria;o__Burkholderiales;f__Alcaligenaceae | 0.37 | 13.33 | 0.66 | 0.74 |
| k__Bacteria;p__Actinobacteria;c__Actinobacteria;o__Bifidobacteriales;f__Bifidobacteriaceae | −0.34 | 13.76 | 0.69 | 0.74 |

* denotes FDR adjusted *p* value

## Pairwise Comparison of Sugar Y, Fiber Y vs. Sugar Y, Fiber N

| Taxon | logFC | logCPM | *p* | *q** |
| --- | --- | --- | --- | --- |
| k__Bacteria;p__Firmicutes;c__Bacilli;o__Lactobacillales;f__Enterococcaceae | −4.11 | 16.55 | 5.24 × 10^−3^ | 0.02 |
| k__Bacteria;p__Firmicutes;c__Bacilli;o__Bacillales;f__Planococcaceae | −4.37 | 10.98 | 3.50 × 10^−4^ | 2.96 × 10^−3^ |
| k__Bacteria;p__Firmicutes;c__Bacilli;o__Bacillales;f__Staphylococcaceae | −3.32 | 16.75 | 3.86 × 10^−4^ | 2.96 × 10^−3^ |
| k__Bacteria;p__Proteobacteria;c__Gammaproteobacteria;o__Enterobacteriales;f__Enterobacteriaceae | 6.77 | 15.64 | 2.50 × 10^−4^ | 2.96 × 10^−3^ |
| k__Bacteria;p__Firmicutes;c__Bacilli;o__Lactobacillales;f__Lactobacillaceae | −0.90 | 16.81 | 0.31 | 0.38 |
| k__Bacteria;p__Firmicutes;c__Clostridia;o__Clostridiales;f__Peptococcaceae | 4.39 | 12.08 | 4.26 × 10^−3^ | 0.02 |
| k__Bacteria;p__Firmicutes;c__Clostridia;o__Clostridiales;f__Lachnospiraceae | 2.90 | 14.52 | 0.02 | 0.05 |
| k__Bacteria;p__Actinobacteria;c__Coriobacteriia;o__Coriobacteriales;f__Coriobacteriaceae | 3.35 | 10.67 | 0.02 | 0.05 |
| k__Bacteria;p__Firmicutes;c__Erysipelotrichi;o__Erysipelotrichales;f__Erysipelotrichaceae | 3.37 | 11.54 | 0.08 | 0.15 |
| k__Bacteria;p__Verrucomicrobia;c__Verrucomicrobiae;o__Verrucomicrobiales;f__Verrucomicrobiaceae | 5.48 | 13.99 | 0.02 | 0.05 |
| k__Bacteria;p__Bacteroidetes;c__Bacteroidia;o__Bacteroidales;f__S24-7 | 1.55 | 15.77 | 0.23 | 0.34 |
| k__Bacteria;p__Firmicutes;c__Bacilli;o__Lactobacillales;f__Streptococcaceae | −0.65 | 17.58 | 0.56 | 0.61 |
| k__Bacteria;p__Firmicutes;c__Clostridia;o__Clostridiales;Other | 1.25 | 13.52 | 0.27 | 0.35 |
| k__Bacteria;p__Proteobacteria;c__Betaproteobacteria;o__Burkholderiales;f__Comamonadaceae | 0.02 | 10.47 | 0.98 | 0.99 |
| k__Bacteria;p__Firmicutes;c__Clostridia;o__Clostridiales;f__Ruminococcaceae | 1.93 | 16.80 | 0.12 | 0.18 |
| k__Bacteria;p__Actinobacteria;c__Actinobacteria;o__Actinomycetales;f__Corynebacteriaceae | −1.62 | 13.52 | 0.05 | 0.10 |
| k__Bacteria;p__Proteobacteria;c__Gammaproteobacteria;o__Pseudomonadales;f__Moraxellaceae | 0.02 | 12.58 | 0.99 | 0.99 |
| Unassigned;Other;Other;Other;Other | 0.89 | 14.86 | 0.28 | 0.35 |
| k__Bacteria;p__Bacteroidetes;c__Bacteroidia;o__Bacteroidales;f__Porphyromonadaceae | 3.16 | 14.65 | 0.03 | 0.07 |
| k__Bacteria;p__Bacteroidetes;c__Bacteroidia;o__Bacteroidales;f__Bacteroidaceae | 2.82 | 15.64 | 0.05 | 0.10 |
| k__Bacteria;p__Proteobacteria;c__Gammaproteobacteria;o__Pasteurellales;f__Pasteurellaceae | 3.46 | 16.37 | 0.09 | 0.15 |
| k__Bacteria;p__Proteobacteria;c__Betaproteobacteria;o__Burkholderiales;f__Alcaligenaceae | 2.81 | 13.63 | 0.10 | 0.16 |
| k__Bacteria;p__Actinobacteria;c__Actinobacteria;o__Bifidobacteriales;f__Bifidobacteriaceae | −0.70 | 14.12 | 0.53 | 0.60 |

* denotes FDR adjusted *p* value

# Differential Analysis at Order Level

## Omnibus Tests

| Taxon | Sugar | | Fiber | | Sugar::Fiber* | |
| --- | --- | --- | --- | --- | --- | --- |
|  | *p* | *q*^†^ | *p* | *q*^†^ | *p* | *q*^†^ |
| k__Bacteria;p__Proteobacteria;c__Gammaproteobacteria;o__Pasteurellales | 2.88 × 10^−3^ | 0.04 | 2.64 × 10^−3^ | 0.04 | 0.45 | 0.70 |
| k__Bacteria;p__Actinobacteria;c__Actinobacteria;o__Actinomycetales | 0.01 | 0.08 | 0.03 | 0.18 | 0.56 | 0.70 |
| k__Bacteria;p__Verrucomicrobia;c__Verrucomicrobiae;o__Verrucomicrobiales | 0.37 | 0.52 | 0.15 | 0.71 | 0.35 | 0.70 |
| k__Bacteria;p__Actinobacteria;c__Actinobacteria;o__Bifidobacteriales | 0.54 | 0.58 | 0.24 | 0.83 | 0.96 | 0.96 |
| k__Bacteria;p__Firmicutes;c__Bacilli;o__Bacillales | 0.26 | 0.45 | 0.33 | 0.87 | 7.02 × 10^−6^ | 9.83 × 10^−5^ |
| k__Bacteria;p__Firmicutes;c__Bacilli;o__Lactobacillales | 0.68 | 0.68 | 0.42 | 0.87 | 0.14 | 0.48 |
| k__Bacteria;p__Proteobacteria;c__Betaproteobacteria;o__Burkholderiales | 0.45 | 0.57 | 0.44 | 0.87 | 0.60 | 0.70 |
| k__Bacteria;p__Actinobacteria;c__Coriobacteriia;o__Coriobacteriales | 0.03 | 0.14 | 0.60 | 1.00 | 0.17 | 0.48 |
| k__Bacteria;p__Proteobacteria;c__Gammaproteobacteria;o__Enterobacteriales | 0.16 | 0.45 | 0.71 | 1.00 | 1.88 × 10^−4^ | 1.32 × 10^−3^ |
| k__Bacteria;p__Proteobacteria;c__Gammaproteobacteria;o__Pseudomonadales | 0.29 | 0.45 | 0.76 | 1.00 | 0.52 | 0.70 |
| k__Bacteria;p__Firmicutes;c__Erysipelotrichi;o__Erysipelotrichales | 0.11 | 0.39 | 0.89 | 1.00 | 0.13 | 0.48 |
| Unassigned;Other;Other;Other | 0.52 | 0.58 | 0.92 | 1.00 | 0.78 | 0.84 |
| k__Bacteria;p__Firmicutes;c__Clostridia;o__Clostridiales | 0.28 | 0.45 | 1.00 | 1.00 | 0.46 | 0.70 |
| k__Bacteria;p__Bacteroidetes;c__Bacteroidia;o__Bacteroidales | 0.28 | 0.45 | 1.00 | 1.00 | 0.34 | 0.70 |

* interaction term of Sugar and Fiber

^†^ denotes FDR adjusted *p* value

## Pairwise Comparison of Sugar N, Fiber Y vs. Sugar N, Fiber N

| Taxon | logFC | logCPM | *p* | *q** |
| --- | --- | --- | --- | --- |
| k__Bacteria;p__Proteobacteria;c__Gammaproteobacteria;o__Pasteurellales | 5.27 | 11.01 | 5.16 × 10^−4^ | 7.23 × 10^−3^ |
| k__Bacteria;p__Actinobacteria;c__Actinobacteria;o__Actinomycetales | −2.28 | 15.96 | 9.45 × 10^−3^ | 0.07 |
| k__Bacteria;p__Verrucomicrobia;c__Verrucomicrobiae;o__Verrucomicrobiales | 2.05 | 12.39 | 0.11 | 0.40 |
| k__Bacteria;p__Actinobacteria;c__Actinobacteria;o__Bifidobacteriales | −1.70 | 14.63 | 0.05 | 0.23 |
| k__Bacteria;p__Firmicutes;c__Bacilli;o__Bacillales | 1.10 | 18.11 | 0.21 | 0.48 |
| k__Bacteria;p__Firmicutes;c__Bacilli;o__Lactobacillales | 0.31 | 19.26 | 0.72 | 0.87 |
| k__Bacteria;p__Proteobacteria;c__Betaproteobacteria;o__Burkholderiales | −0.22 | 13.22 | 0.78 | 0.87 |
| k__Bacteria;p__Actinobacteria;c__Coriobacteriia;o__Coriobacteriales | −0.23 | 10.86 | 0.81 | 0.87 |
| k__Bacteria;p__Proteobacteria;c__Gammaproteobacteria;o__Enterobacteriales | −0.54 | 11.58 | 0.54 | 0.84 |
| k__Bacteria;p__Proteobacteria;c__Gammaproteobacteria;o__Pseudomonadales | −0.04 | 13.40 | 0.96 | 0.96 |
| k__Bacteria;p__Firmicutes;c__Erysipelotrichi;o__Erysipelotrichales | −1.04 | 10.91 | 0.24 | 0.48 |
| Unassigned;Other;Other;Other | 0.31 | 14.56 | 0.68 | 0.87 |
| k__Bacteria;p__Firmicutes;c__Clostridia;o__Clostridiales | −0.88 | 17.00 | 0.29 | 0.51 |
| k__Bacteria;p__Bacteroidetes;c__Bacteroidia;o__Bacteroidales | −1.08 | 16.51 | 0.22 | 0.48 |

* denotes FDR adjusted *p* value

## Pairwise Comparison of Sugar Y, Fiber N vs. Sugar N, Fiber N

| Taxon | logFC | logCPM | *p* | *q** |
| --- | --- | --- | --- | --- |
| k__Bacteria;p__Proteobacteria;c__Gammaproteobacteria;o__Pasteurellales | 6.07 | 11.85 | 1.66 × 10^−3^ | 0.01 |
| k__Bacteria;p__Actinobacteria;c__Actinobacteria;o__Actinomycetales | −2.64 | 15.77 | 1.20 × 10^−3^ | 0.01 |
| k__Bacteria;p__Verrucomicrobia;c__Verrucomicrobiae;o__Verrucomicrobiales | −1.38 | 10.64 | 0.56 | 0.71 |
| k__Bacteria;p__Actinobacteria;c__Actinobacteria;o__Bifidobacteriales | −1.12 | 15.36 | 0.33 | 0.46 |
| k__Bacteria;p__Firmicutes;c__Bacilli;o__Bacillales | 0.06 | 17.65 | 0.94 | 0.96 |
| k__Bacteria;p__Firmicutes;c__Bacilli;o__Lactobacillales | 0.14 | 19.26 | 0.87 | 0.96 |
| k__Bacteria;p__Proteobacteria;c__Betaproteobacteria;o__Burkholderiales | −0.96 | 12.91 | 0.33 | 0.46 |
| k__Bacteria;p__Actinobacteria;c__Coriobacteriia;o__Coriobacteriales | −2.69 | 10.16 | 0.07 | 0.23 |
| k__Bacteria;p__Proteobacteria;c__Gammaproteobacteria;o__Enterobacteriales | −2.01 | 11.24 | 0.04 | 0.18 |
| k__Bacteria;p__Proteobacteria;c__Gammaproteobacteria;o__Pseudomonadales | −1.17 | 12.96 | 0.18 | 0.44 |
| k__Bacteria;p__Firmicutes;c__Erysipelotrichi;o__Erysipelotrichales | −2.16 | 10.71 | 0.19 | 0.44 |
| Unassigned;Other;Other;Other | −0.04 | 14.40 | 0.96 | 0.96 |
| k__Bacteria;p__Firmicutes;c__Clostridia;o__Clostridiales | −1.35 | 17.00 | 0.24 | 0.46 |
| k__Bacteria;p__Bacteroidetes;c__Bacteroidia;o__Bacteroidales | −1.26 | 16.53 | 0.31 | 0.46 |

* denotes FDR adjusted *p* value

## Pairwise Comparison of Sugar Y, Fiber Y vs. Sugar N, Fiber Y

| Taxon | logFC | logCPM | *p* | *q** |
| --- | --- | --- | --- | --- |
| k__Bacteria;p__Proteobacteria;c__Gammaproteobacteria;o__Pasteurellales | 4.24 | 16.16 | 0.03 | 0.09 |
| k__Bacteria;p__Actinobacteria;c__Actinobacteria;o__Actinomycetales | −2.67 | 14.10 | 3.52 × 10^−3^ | 0.02 |
| k__Bacteria;p__Verrucomicrobia;c__Verrucomicrobiae;o__Verrucomicrobiales | 0.67 | 14.04 | 0.59 | 0.91 |
| k__Bacteria;p__Actinobacteria;c__Actinobacteria;o__Bifidobacteriales | −0.68 | 13.95 | 0.48 | 0.91 |
| k__Bacteria;p__Firmicutes;c__Bacilli;o__Bacillales | −4.53 | 17.43 | 2.39 × 10^−6^ | 3.34 × 10^−5^ |
| k__Bacteria;p__Firmicutes;c__Bacilli;o__Lactobacillales | −1.60 | 18.65 | 0.10 | 0.22 |
| k__Bacteria;p__Proteobacteria;c__Betaproteobacteria;o__Burkholderiales | −6.86 × 10^−3^ | 13.84 | 0.99 | 0.99 |
| k__Bacteria;p__Actinobacteria;c__Coriobacteriia;o__Coriobacteriales | −0.36 | 11.25 | 0.70 | 0.96 |
| k__Bacteria;p__Proteobacteria;c__Gammaproteobacteria;o__Enterobacteriales | 4.85 | 15.34 | 2.58 × 10^−3^ | 0.02 |
| k__Bacteria;p__Proteobacteria;c__Gammaproteobacteria;o__Pseudomonadales | −1.69 | 13.36 | 0.08 | 0.22 |
| k__Bacteria;p__Firmicutes;c__Erysipelotrichi;o__Erysipelotrichales | 0.62 | 11.47 | 0.56 | 0.91 |
| Unassigned;Other;Other;Other | 0.19 | 14.92 | 0.82 | 0.96 |
| k__Bacteria;p__Firmicutes;c__Clostridia;o__Clostridiales | −0.05 | 17.27 | 0.96 | 0.99 |
| k__Bacteria;p__Bacteroidetes;c__Bacteroidia;o__Bacteroidales | 0.27 | 17.03 | 0.78 | 0.96 |

* denotes FDR adjusted *p* value

## Pairwise Comparison of Sugar Y, Fiber Y vs. Sugar Y, Fiber N

| Taxon | logFC | logCPM | *p* | *q** |
| --- | --- | --- | --- | --- |
| k__Bacteria;p__Proteobacteria;c__Gammaproteobacteria;o__Pasteurellales | 4.14 | 16.82 | 0.05 | 0.11 |
| k__Bacteria;p__Actinobacteria;c__Actinobacteria;o__Actinomycetales | −2.39 | 14.08 | 7.57 × 10^−3^ | 0.03 |
| k__Bacteria;p__Verrucomicrobia;c__Verrucomicrobiae;o__Verrucomicrobiales | 5.51 | 13.30 | 7.61 × 10^−3^ | 0.03 |
| k__Bacteria;p__Actinobacteria;c__Actinobacteria;o__Bifidobacteriales | −0.64 | 14.15 | 0.56 | 0.62 |
| k__Bacteria;p__Firmicutes;c__Bacilli;o__Bacillales | −4.74 | 17.79 | 1.69 × 10^−5^ | 2.36 × 10^−4^ |
| k__Bacteria;p__Firmicutes;c__Bacilli;o__Lactobacillales | −1.71 | 19.10 | 0.10 | 0.18 |
| k__Bacteria;p__Proteobacteria;c__Betaproteobacteria;o__Burkholderiales | 1.21 | 13.18 | 0.15 | 0.21 |
| k__Bacteria;p__Actinobacteria;c__Coriobacteriia;o__Coriobacteriales | 3.07 | 10.22 | 0.01 | 0.03 |
| k__Bacteria;p__Proteobacteria;c__Gammaproteobacteria;o__Enterobacteriales | 5.93 | 15.61 | 1.22 × 10^−3^ | 8.51 × 10^−3^ |
| k__Bacteria;p__Proteobacteria;c__Gammaproteobacteria;o__Pseudomonadales | −0.50 | 12.97 | 0.62 | 0.62 |
| k__Bacteria;p__Firmicutes;c__Erysipelotrichi;o__Erysipelotrichales | 2.48 | 10.82 | 0.15 | 0.21 |
| Unassigned;Other;Other;Other | 0.46 | 15.24 | 0.62 | 0.62 |
| k__Bacteria;p__Firmicutes;c__Clostridia;o__Clostridiales | 1.41 | 16.53 | 0.17 | 0.21 |
| k__Bacteria;p__Bacteroidetes;c__Bacteroidia;o__Bacteroidales | 1.87 | 16.26 | 0.10 | 0.18 |

* denotes FDR adjusted *p* value

# Differential Analysis at Class Level

## Omnibus Tests

| Taxon | Sugar | | Fiber | | Sugar::Fiber* | |
| --- | --- | --- | --- | --- | --- | --- |
|  | *p* | *q*^†^ | *p* | *q*^†^ | *p* | *q*^†^ |
| k__Bacteria;p__Actinobacteria;c__Coriobacteriia | 7.57 × 10^−4^ | 8.33 × 10^−3^ | 0.59 | 0.68 | 8.31 × 10^−3^ | 0.03 |
| k__Bacteria;p__Firmicutes;c__Erysipelotrichi | 6.57 × 10^−3^ | 0.02 | 0.35 | 0.61 | 0.02 | 0.03 |
| k__Bacteria;p__Bacteroidetes;c__Bacteroidia | 7.55 × 10^−3^ | 0.02 | 0.21 | 0.48 | 0.01 | 0.03 |
| k__Bacteria;p__Firmicutes;c__Clostridia | 8.54 × 10^−3^ | 0.02 | 0.21 | 0.48 | 0.04 | 0.07 |
| k__Bacteria;p__Proteobacteria;c__Gammaproteobacteria | 0.01 | 0.03 | 0.69 | 0.69 | 0.41 | 0.50 |
| k__Bacteria;p__Firmicutes;c__Bacilli | 0.04 | 0.07 | 0.39 | 0.61 | 1.35 × 10^−3^ | 0.01 |
| Unassigned;Other;Other | 0.04 | 0.07 | 0.57 | 0.68 | 0.46 | 0.50 |
| k__Bacteria;p__Verrucomicrobia;c__Verrucomicrobiae | 0.06 | 0.09 | 0.22 | 0.48 | 0.09 | 0.13 |
| k__Bacteria;p__Actinobacteria;c__Actinobacteria | 0.12 | 0.15 | 2.50 × 10^−3^ | 0.03 | 0.93 | 0.93 |
| k__Bacteria;p__Proteobacteria;c__Alphaproteobacteria | 0.14 | 0.16 | 0.14 | 0.48 | 9.88 × 10^−3^ | 0.03 |
| k__Bacteria;p__Proteobacteria;c__Betaproteobacteria | 0.34 | 0.34 | 0.61 | 0.68 | 0.36 | 0.49 |

* interaction term of Sugar and Fiber

^†^ denotes FDR adjusted *p* value

## Pairwise Comparison of Sugar N, Fiber Y vs. Sugar N, Fiber N

| Taxon | logFC | logCPM | *p* | *q** |
| --- | --- | --- | --- | --- |
| k__Bacteria;p__Actinobacteria;c__Coriobacteriia | 0.12 | 10.67 | 0.89 | 0.89 |
| k__Bacteria;p__Firmicutes;c__Erysipelotrichi | −0.55 | 10.94 | 0.51 | 0.70 |
| k__Bacteria;p__Bacteroidetes;c__Bacteroidia | −0.28 | 16.47 | 0.74 | 0.82 |
| k__Bacteria;p__Firmicutes;c__Clostridia | −0.30 | 16.88 | 0.71 | 0.82 |
| k__Bacteria;p__Proteobacteria;c__Gammaproteobacteria | 0.80 | 14.11 | 0.30 | 0.55 |
| k__Bacteria;p__Firmicutes;c__Bacilli | 1.07 | 20.12 | 0.21 | 0.47 |
| Unassigned;Other;Other | 0.90 | 14.63 | 0.21 | 0.47 |
| k__Bacteria;p__Verrucomicrobia;c__Verrucomicrobiae | 2.11 | 12.50 | 0.11 | 0.47 |
| k__Bacteria;p__Actinobacteria;c__Actinobacteria | −1.64 | 16.25 | 0.01 | 0.16 |
| k__Bacteria;p__Proteobacteria;c__Alphaproteobacteria | 1.08 | 11.69 | 0.21 | 0.47 |
| k__Bacteria;p__Proteobacteria;c__Betaproteobacteria | 0.49 | 13.20 | 0.48 | 0.70 |

* denotes FDR adjusted *p* value

## Pairwise Comparison of Sugar Y, Fiber N vs. Sugar N, Fiber N

| Taxon | logFC | logCPM | *p* | *q** |
| --- | --- | --- | --- | --- |
| k__Bacteria;p__Actinobacteria;c__Coriobacteriia | −2.06 | 9.83 | 0.11 | 0.59 |
| k__Bacteria;p__Firmicutes;c__Erysipelotrichi | −1.65 | 10.47 | 0.20 | 0.59 |
| k__Bacteria;p__Bacteroidetes;c__Bacteroidia | −0.80 | 16.14 | 0.41 | 0.59 |
| k__Bacteria;p__Firmicutes;c__Clostridia | −0.74 | 16.58 | 0.43 | 0.59 |
| k__Bacteria;p__Proteobacteria;c__Gammaproteobacteria | 0.53 | 13.88 | 0.58 | 0.71 |
| k__Bacteria;p__Firmicutes;c__Bacilli | 1.02 | 19.96 | 0.25 | 0.59 |
| Unassigned;Other;Other | 0.86 | 14.53 | 0.33 | 0.59 |
| k__Bacteria;p__Verrucomicrobia;c__Verrucomicrobiae | −1.26 | 10.50 | 0.39 | 0.59 |
| k__Bacteria;p__Actinobacteria;c__Actinobacteria | −1.09 | 16.29 | 0.21 | 0.59 |
| k__Bacteria;p__Proteobacteria;c__Alphaproteobacteria | 0.09 | 11.03 | 0.94 | 0.94 |
| k__Bacteria;p__Proteobacteria;c__Betaproteobacteria | −0.32 | 12.68 | 0.76 | 0.83 |

* denotes FDR adjusted *p* value

## Pairwise Comparison of Sugar Y, Fiber Y vs. Sugar N, Fiber Y

| Taxon | logFC | logCPM | *p* | *q** |
| --- | --- | --- | --- | --- |
| k__Bacteria;p__Actinobacteria;c__Coriobacteriia | −0.09 | 11.04 | 0.91 | 0.98 |
| k__Bacteria;p__Firmicutes;c__Erysipelotrichi | 0.56 | 11.21 | 0.54 | 0.93 |
| k__Bacteria;p__Bacteroidetes;c__Bacteroidia | 0.46 | 16.75 | 0.56 | 0.93 |
| k__Bacteria;p__Firmicutes;c__Clostridia | 0.04 | 17.03 | 0.95 | 0.98 |
| k__Bacteria;p__Proteobacteria;c__Gammaproteobacteria | 3.34 | 17.11 | 4.38 × 10^−3^ | 0.02 |
| k__Bacteria;p__Firmicutes;c__Bacilli | −2.45 | 19.63 | 7.81 × 10^−3^ | 0.03 |
| Unassigned;Other;Other | 0.02 | 15.08 | 0.98 | 0.98 |
| k__Bacteria;p__Verrucomicrobia;c__Verrucomicrobiae | 0.61 | 13.82 | 0.59 | 0.93 |
| k__Bacteria;p__Actinobacteria;c__Actinobacteria | −1.35 | 15.02 | 0.06 | 0.16 |
| k__Bacteria;p__Proteobacteria;c__Alphaproteobacteria | −1.92 | 11.65 | 9.70 × 10^−4^ | 0.01 |
| k__Bacteria;p__Proteobacteria;c__Betaproteobacteria | −0.06 | 13.67 | 0.91 | 0.98 |

* denotes FDR adjusted *p* value

## Pairwise Comparison of Sugar Y, Fiber Y vs. Sugar Y, Fiber N

| Taxon | logFC | logCPM | *p* | *q** |
| --- | --- | --- | --- | --- |
| k__Bacteria;p__Actinobacteria;c__Coriobacteriia | 2.80 | 10.23 | 0.03 | 0.09 |
| k__Bacteria;p__Firmicutes;c__Erysipelotrichi | 1.92 | 10.71 | 0.24 | 0.32 |
| k__Bacteria;p__Bacteroidetes;c__Bacteroidia | 1.57 | 16.36 | 0.16 | 0.25 |
| k__Bacteria;p__Firmicutes;c__Clostridia | 0.92 | 16.62 | 0.36 | 0.38 |
| k__Bacteria;p__Proteobacteria;c__Gammaproteobacteria | 3.81 | 17.15 | 2.95 × 10^−3^ | 0.02 |
| k__Bacteria;p__Firmicutes;c__Bacilli | −3.50 | 20.74 | 2.87 × 10^−3^ | 0.02 |
| Unassigned;Other;Other | −0.86 | 15.52 | 0.38 | 0.38 |
| k__Bacteria;p__Verrucomicrobia;c__Verrucomicrobiae | 4.43 | 12.94 | 0.02 | 0.07 |
| k__Bacteria;p__Actinobacteria;c__Actinobacteria | −1.97 | 15.50 | 0.04 | 0.09 |
| k__Bacteria;p__Proteobacteria;c__Alphaproteobacteria | −1.59 | 11.01 | 0.10 | 0.18 |
| k__Bacteria;p__Proteobacteria;c__Betaproteobacteria | 0.79 | 13.21 | 0.37 | 0.38 |

* denotes FDR adjusted *p* value

# Differential Analysis at Phylum Level

## Omnibus Tests

| Taxon | Sugar | | Fiber | | Sugar::Fiber* | |
| --- | --- | --- | --- | --- | --- | --- |
|  | *p* | *q*^†^ | *p* | *q*^†^ | *p* | *q*^†^ |
| k__Bacteria;p__Proteobacteria | 0.50 | 0.56 | 0.40 | 0.80 | 0.02 | 0.10 |
| k__Bacteria;p__Bacteroidetes | 0.12 | 0.25 | 0.85 | 0.98 | 0.05 | 0.14 |
| k__Bacteria;p__Verrucomicrobia | 0.12 | 0.25 | 0.04 | 0.12 | 0.07 | 0.14 |
| k__Bacteria;p__Firmicutes | 0.38 | 0.56 | 0.98 | 0.98 | 0.36 | 0.54 |
| k__Bacteria;p__Actinobacteria | 0.08 | 0.25 | 0.04 | 0.12 | 0.51 | 0.61 |
| Unassigned;Other | 0.56 | 0.56 | 0.89 | 0.98 | 0.63 | 0.63 |

* interaction term of Sugar and Fiber

^†^ denotes FDR adjusted *p* value

## Pairwise Comparison of Sugar N, Fiber Y vs. Sugar N, Fiber N

| Taxon | logFC | logCPM | *p* | *q** |
| --- | --- | --- | --- | --- |
| k__Bacteria;p__Proteobacteria | 1.02 | 14.94 | 0.19 | 0.29 |
| k__Bacteria;p__Bacteroidetes | 0.33 | 16.32 | 0.58 | 0.58 |
| k__Bacteria;p__Verrucomicrobia | 2.87 | 12.78 | 2.00 × 10^−3^ | 0.01 |
| k__Bacteria;p__Firmicutes | 0.93 | 19.97 | 0.14 | 0.28 |
| k__Bacteria;p__Actinobacteria | −1.05 | 16.10 | 0.10 | 0.28 |
| Unassigned;Other | 0.95 | 14.55 | 0.25 | 0.30 |

* denotes FDR adjusted *p* value

## Pairwise Comparison of Sugar Y, Fiber N vs. Sugar N, Fiber N

| Taxon | logFC | logCPM | *p* | *q** |
| --- | --- | --- | --- | --- |
| k__Bacteria;p__Proteobacteria | 0.41 | 14.88 | 0.59 | 0.75 |
| k__Bacteria;p__Bacteroidetes | −1.27 | 16.48 | 0.29 | 0.75 |
| k__Bacteria;p__Verrucomicrobia | −1.78 | 10.81 | 0.46 | 0.75 |
| k__Bacteria;p__Firmicutes | 0.24 | 19.59 | 0.63 | 0.75 |
| k__Bacteria;p__Actinobacteria | −1.64 | 16.49 | 0.02 | 0.11 |
| Unassigned;Other | 0.15 | 14.28 | 0.80 | 0.80 |

* denotes FDR adjusted *p* value

## Pairwise Comparison of Sugar Y, Fiber Y vs. Sugar N, Fiber Y

| Taxon | logFC | logCPM | *p* | *q** |
| --- | --- | --- | --- | --- |
| k__Bacteria;p__Proteobacteria | 3.19 | 17.55 | 1.82 × 10^−4^ | 1.09 × 10^−3^ |
| k__Bacteria;p__Bacteroidetes | 1.05 | 17.30 | 0.20 | 0.33 |
| k__Bacteria;p__Verrucomicrobia | 1.13 | 14.41 | 0.22 | 0.33 |
| k__Bacteria;p__Firmicutes | −0.28 | 19.21 | 0.70 | 0.70 |
| k__Bacteria;p__Actinobacteria | −0.70 | 14.94 | 0.44 | 0.53 |
| Unassigned;Other | 1.17 | 14.92 | 0.20 | 0.33 |

* denotes FDR adjusted *p* value

## Pairwise Comparison of Sugar Y, Fiber Y vs. Sugar Y, Fiber N

| Taxon | logFC | logCPM | *p* | *q** |
| --- | --- | --- | --- | --- |
| k__Bacteria;p__Proteobacteria | 3.33 | 17.37 | 4.77 × 10^−4^ | 1.43 × 10^−3^ |
| k__Bacteria;p__Bacteroidetes | 2.18 | 16.86 | 0.02 | 0.04 |
| k__Bacteria;p__Verrucomicrobia | 5.38 | 13.75 | 2.96 × 10^−5^ | 1.78 × 10^−4^ |
| k__Bacteria;p__Firmicutes | −0.90 | 19.42 | 0.28 | 0.42 |
| k__Bacteria;p__Actinobacteria | −0.99 | 14.96 | 0.35 | 0.42 |
| Unassigned;Other | 0.75 | 14.90 | 0.47 | 0.47 |

* denotes FDR adjusted *p* value
